# ShatterProof: operational detection and quantification of chromothripsis

**DOI:** 10.1186/1471-2105-15-78

**Published:** 2014-03-19

**Authors:** Shaylan K Govind, Amin Zia, Pablo H Hennings-Yeomans, John D Watson, Michael Fraser, Catalina Anghel, Alexander W Wyatt, Theodorus van der Kwast, Colin C Collins, John D McPherson, Robert G Bristow, Paul C Boutros

**Affiliations:** 1 Ontario Institute for Cancer Research, M5G 0A3, Toronto, Canada; 2 Vancouver Prostate Centre and Department of Urologic Sciences, University of British Columbia, Vancouver, BC, Canada; 3 STTARR Innovation Program, Toronto, Ontario, Canada; 4 Radiation Medicine Program, Ontario Cancer Institute, Toronto, Ontario, Canada; 5 Department of Medical Biophysics, University of Toronto, Toronto, Ontario, Canada; 6 Department of Pathology, University Health Network, Toronto, Ontario, Canada; 7 Department of Pharmocology and Toxicology, University of Toronto, Toronto, Ontario, Canada

**Keywords:** Chromothripsis, Complex genomic rearrangement, Next generation sequencing, High throughput sequencing, Perl, Bioinformatics

## Abstract

**Background:**

Chromothripsis, a newly discovered type of complex genomic rearrangement, has been implicated in the evolution of several types of cancers. To date, it has been described in bone cancer, SHH-medulloblastoma and acute myeloid leukemia, amongst others, however there are still no formal or automated methods for detecting or annotating it in high throughput sequencing data. As such, findings of chromothripsis are difficult to compare and many cases likely escape detection altogether.

**Results:**

We introduce ShatterProof, a software tool for detecting and quantifying chromothriptic events. ShatterProof takes structural variation calls (translocations, copy-number variations, short insertions and loss of heterozygosity) produced by any algorithm and using an operational definition of chromothripsis performs robust statistical tests to accurately predict the presence and location of chromothriptic events. Validation of our tool was conducted using clinical data sets including matched normal, prostate cancer samples in addition to the colorectal cancer and SCLC data sets used in the original description of chromothripsis.

**Conclusions:**

ShatterProof is computationally efficient, having low memory requirements and near linear computation time. This allows it to become a standard component of sequencing analysis pipelines, enabling researchers to routinely and accurately assess samples for chromothripsis. Source code and documentation can be found at http://search.cpan.org/~sgovind/Shatterproof.

## Background

Chromothripsis is a type of complex genomic rearrangement first characterized in 2011 [1]. Stephens *et al.* described a phenomenon wherein a chromosome appeared to shatter into hundreds of pieces, then haphazardly stitch itself back together. The resulting chromosomes possess a very high number of structural variations (SVs) including duplications, deletions and translocations [1]. The most striking feature of these derivative chromosomes is pronounced clustering of translocations and copy number aberrations to specific regions.

The exact mechanisms driving chromosome shattering remain unknown, however aberrant mitosis producing micronuclei [2] and premature chromosome compaction (PCC) [2,3] have been implicated. Similarly, the mechanisms driving the stitching process have yet to be determined, but recent work suggests that DNA repair mechanisms such as non-homologous end joining (NHEJ), fork stalling and template switching (FoSTeS) and micro-homology mediated break induced repair (MMBIR) are involved [4,5].

Recent genome sequencing studies have identified several classes of complex genomic rearrangements that appear to be derived from a single catastrophic event rather than numerous incremental steps. In [6], Zhang *et al.* discuss these phenomena, summarize current models, and consider the impact of massive chromosomal change on the development of diseases such as cancer. Since the evolutionary processes that cause chromothripsis are unique, it is likely that chromothriptic genomes produce phenotypes quite distinct from genomes whose SVs arise gradually over time. As such, identifying patients with signs of chromothripsis may lead to improvements in classification and thus help guide clinical decision making.

Studies have indicated that there exists a certain amount of heterogeneity with respect to the genotypes of genomes resulting from chromothripsis. The complexity of the variations found in chromothriptic genomes vary somewhat between reported cases, however studies have also revealed a set of hallmark characteristics that are present in almost all cases, including: 

1. Localization of structural variations to a few chromosomes [1,7]

2. Localization of structural variations within a chromosome to a specific region (*e.g.* the distal arm or telometric region [1,4])

3. A low number of copy-number states, usually only two. Typically, one of these is the normal copy number (CN) state and the other is a deletion state indicating fragments of the genome that have been lost [1]

4. A high number of transitions from the normal copy-number state to the aberrant one [1]

5. In regions of normal copy-number, heterozygosity is preserved [1,3,4]

6. Chromosomal translocations demonstrate a high level of clustering in particular regions [1-4,7]

7. Short insertions at translocation breakpoints [1,3,4,7], indicative of both NHEJ and MMBIR (not clear if these are template or non-template insertions)

8. The TP53 gene is nonsynonymously mutated [3]

These hallmarks have been discovered in 2%-3% of all cancers [8] and in approximately 25% of bone cancers [1]. More recent investigations have discovered chromothripsis in medulloblastomas [9], acute myeloid leukemia [3], in 5% of prostate cancers [8,10] and in 18% of neuroblastomas [11]. Additionally, chromothripsis has been discovered in the germ lines of a number of individuals suffering from developmental and congenital defects [12,13]. Thus far, chromothripsis has been primarily identified using *ad hoc* methods which annotate a chromosome as having undergone chromothripsis if one or two of the chromothriptic hallmarks are detected. However, these methods are only employed after chromosomes are identified to be suspicious, typically by visual inspection. Additionally, the criteria used to annotate a chromothriptic event vary between investigators, making comparisons cumbersome and casting doubts on relative frequency statistics. As such, there is an urgent need for standardized and unbiased metrics to quantify chromothriptic events.

We present a new approach, called ShatterProof, that aims to address these shortcomings. ShatterProof enables the efficient identification and quantification of chromothriptic events in next-generation sequencing data without the need to pre-screen for suspicious samples. ShatterProof quantifies the degree to which chromothriptic hallmarks are expressed. The precise definition of chromothripsis including all of its features remains a contentious topic and until a single clear definition is decided upon, analyzing sequencing data with respect to the current set of hallmarks will enable further investigation and improved detection.

When provided with SV calls from next generation sequencing data, ShatterProof generates metrics describing each of the chromothriptic hallmarks and analyzes these to identify locations in the genome where chromothriptic events have most likely occurred.

A standardized report that is both human readable and machine parseable is created. This report contains all of the metrics and probability values for potential chromothriptic events, enabling easier analysis and comparison across studies.

## Implementation

ShatterProof is implemented as a Perl module that processes input files and produces output files in both tab-delimited and YAML format [14]. Perl version 5.10 or greater is required. ShatterProof was designed to be highly modular. This allows for sub-methods to be easily re-used and enabled robust testing of all stages of the pipeline. Additionally, due to the very large range of input values, many design decisions focused on error-resilience. Pre- and post-condition checking were employed to ensure correctness of calculations and processing. Unit and regression testing consisting of 65 test cases ensure that correct behaviour of input parsing and score-generating methods is maintained.

### Input and workflow

ShatterProof bases its analysis of genomic data on calls of SVs, including translocations, copy number variations (CNV), loss of heterozygosity (LOH) and short insertions. Currently, there is no gold standard tool for producing any of these. Accordingly, ShatterProof has been designed to work with output produced by any SV tool. In this way ShatterProof will always be able to leverage the improving capabilities of newer identification tools in a seamless way. Because different tools produce output in a different format, ShatterProof requires that these be converted into simple tab delimited input file formats that contain only the information needed for chromothriptic analysis. Input file formats were specified for each type of SV that would be analyzed: translocations, CNVs, LOH events and short insertions. Scripts to convert the output of some common SV tools to the input file formats required by ShatterProof are provided with the ShatterProof distribution. Translocations are recorded in.spt files which contain the following fields: 

1. source chromosome

2. start position on source chromosome

3. end position on source chromosome

4. destination chromosome

5. start position on destination chromosome

6. end position on destination chromosome

7. quality of the call (value between 0 and 1)

CNVs are recorded in.spc files which contain the following fields: 

1. chromosome

2. start position

3. end position

4. copy number

5. quality of the call (value between 0 and 1)

LOH events are recorded in.spl files which contain the following fields: 

1. chromosome

2. start position

3. end position

4. quality of the call (value between 0 and 1)

Additionally, ShatterProof accepts insertion calls in VCF version 4.0 [15] files. ShatterProof analyzes the CHROM, POS, REF, and ALT fields of these files.

Examples of each input file format can be seen in Additional file 1.

**Figure 1:**
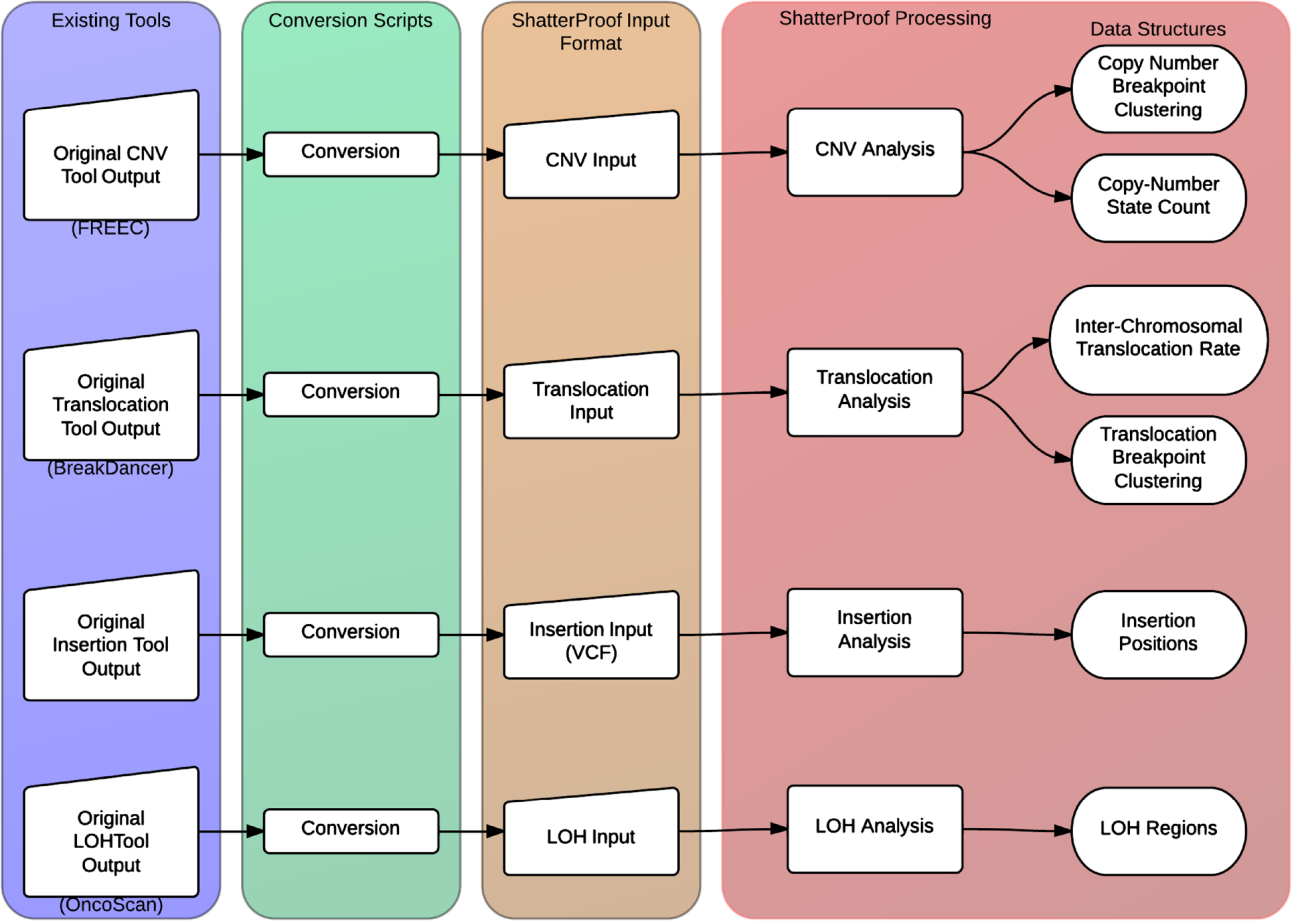
**ShatterProof input parsing workflow.** Original output from existing tools is converted to the ShatterProof input file formats using scripts. ShatterProof then reads and parses the data in these input files into efficient data structures.

Figure 1 illustrates the ShatterProof work flow that produces the data structures containing the processed input data. ShatterProof will generate the most accurate results if all four types of input files are provided, but can accommodate significant missing input data: only translocations and CNVs are required.

The data structures containing the SV calls, produced from parsing the input files, are then subjected to a sliding window analysis to compute metrics of SV density across the genome. The size of the sliding window is a user definable parameter. A smaller window increases base pair resolution but also increases running time and total memory consumption. A default window size of 10 Mbp where each window overlaps with the previous one on 9.99 Mbps was selected as it produced calls with useful resolution in a reasonable running time (see Performance for details). The metrics produced from the sliding window analysis are used to identify highly mutated regions by comparing region-specific SV density to genome-wide density using a z-scale approach. A chromothripsis score is then calculated for each highly mutated region as outlined below.

### Scoring

For each highly mutated region, a numerical score between 0 and 1 is calculated to indicate the likelihood that chromothripsis has occurred. This score is:

(1) $$\overset{N}{\underset{n=1}{\sum }}(\text{hallmark}\;{\text{weight}}_{n})(\text{hallmark}\;{\text{score}}_{n})$$

Where, *N* is the total number of hallmarks, *hallmark weight*_*n*_ is a numerical representation of how significantly the hallmark indicates chromothripsis (described below) and *hallmark score*_*n*_ is a measure of the degree to which the region exhibits this hallmark.

#### Hallmark weights

Certain hallmarks are more indicative of chromothripsis than others. For example, the localization of translocations and CNVs to a specific region in the chromosome is a stronger indicator than is the presence of a TP53 mutation or the presence of short insertions at translocation breakpoints. Indeed, localization of translocations and CNVs to a specific region of a chromosome was found in all cases of chromothripsis whereas this was not the case for the TP53 mutations [1,3]. To account for the varying significance of hallmarks when generating the chromothripsis score, multi-criteria analysis (MCA) [16] was used to calculate hallmark weightings. This process produces values that quantify the relative importance of qualitative attributes. LibreOffice (v3.5.4.2) was used to perform the MCA calculations (see Additional file 2 for source spreadsheet). Intermediate values can be found in Appendix A (Additional file 3). The resulting weights are shown in Table 1.

**Table 1:** MCA calculated hallmark weights

**Hallmark**	**Weighting**
Genome localization	0.1145
Chromosome localization	0.1697
Copy-number aberrations	0.2724
Translocation localization	0.2724
Retention of heterozgosity	0.0648
Presences of insertions at translocation breakpoints	0.0657
Presences of TP53 mutations	0.0406
	**Sum**: 1.0000

The value of these weightings can be user customized depending on the relative quality of input data that is being given to ShatterProof. For example, if very high quality translocation calls are produced, a user can choose to increase the weight of the translocation hallmark so that it makes a larger contribution to the final score.

#### Hallmark scores

**Figure 2:**
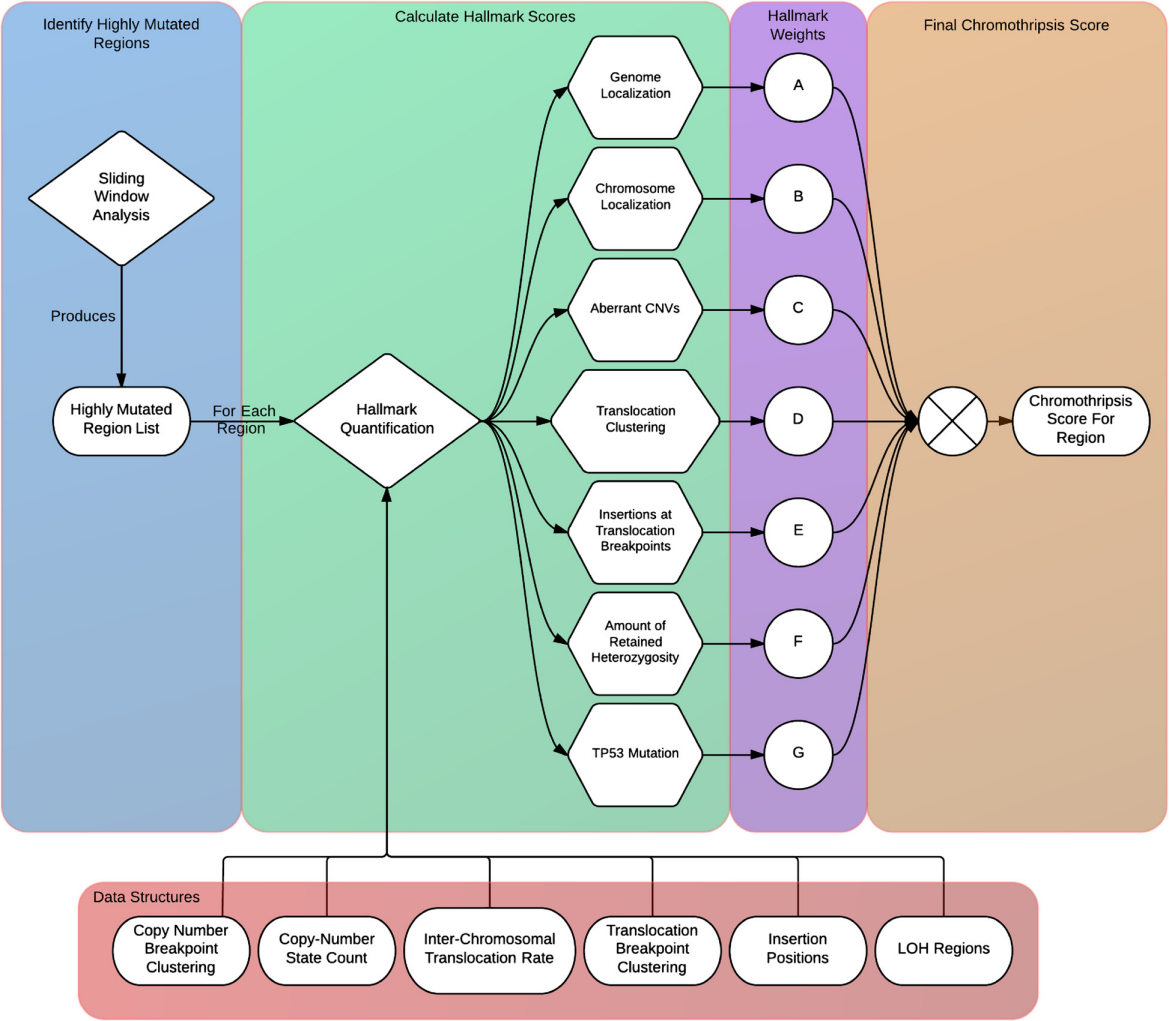
**Suspect region identification and score calculation.** Once ShatterProof has parsed all input data, a sliding window analysis identifies genomic regions which are heavily mutated. Note that the window size is user definable. ShatterProof then produces a chromothripsis score for each of these regions, by analyzing all SV data corresponding to it. The analysis produces hallmark scores which represent how significantly the region exhibits each hallmark,which are then scaled by their respective MCA weighting and summed to produce a chromothripsis score.

ShatterProof calculates hallmark scores for each highly mutated region by performing statistical analysis on the SV data for that region. Each score is in [0,1], where 1 indicates that the hallmark is strongly represented in the region and 0 indicates the hallmark is not represented at all. Figure 2 illustrates the ShatterProof work flow that produces the hallmark and final chromothripsis scores for each highly mutated region. The following sections describe the equations used to calculate the score for each chromothriptic hallmark.

#### Genome localization

The density of SVs in each chromosome is determined by summing the number of translocations breakpoints and CNV breakpoints, scaled by the total chromosome length. A translocation breakpoint is defined as one end of a translocated region, therefore all translocations are comprised of four breakpoints, two from the originating chromosome and two from the destination chromosome. A CNV breakpoint is defined as one end of an amplified or deleted region, therefore all CNVs are comprised of two breakpoints. If translocations or CNVs occurs at the end of a chromosome (*e.g.* loss of chromosome arm) a breakpoint is inserted after the last base pair of the chromosome. If a SV occurs at the start of a chromosome, a breakpoint is placed before the first base pair of the chromosome. The genome localization score for a highly mutated region is calculated from the z-score for the SV density of the chromosome in which it is found. This z-score is converted to a right-tailed p-value via the standard normal distribution, giving a genome localization score as:

(2) $$\text{Genome}\;\text{Localization}\;\text{Score}=\frac{0.5-p}{0.5}$$

To avoid multiple-testing, scores will only be calculated for chromosomes whose SV density is greater than the mean of all chromosomes, resulting in z-scores always being greater than 0. As such, the right-tailed p-value (*p*) will have a maximum value of 0.5 and a minimum value of 0. A genome localization score near 1 (resulting from a high z-score) indicates that the SV density of the suspect chromosome is much greater than the mean of the SV densities of all the chromosomes, and a score near 0 (resulting from a low z-score) indicates that the SV density of the suspect chromosome is close to the mean and thus is insignificant.

#### Chromosome localization

To calculate the chromosome localization score for a highly mutated region, the SV density of the region is compared to the overall SV density of the chromosome using Pearson’s chi-squared test with one degree of freedom. The test statistic is calculated as:

(3) $$\chi^{2}=\frac{{\left(\text{SV}\;{\text{Density}}_{\text{region}}-\text{SV}\;{\text{Density}}_{\text{chromosome}}\right)}^{2}}{\text{SV}\;{\text{Density}}_{\text{chromosome}}}$$

#### Low number of copy-number states and high number of copy-number state oscillations

Chromothripsis is characterized by a low number of different aberrant CN states and a high number of CN state oscillations [1,5,6]. To quantify this, a higher CNV score is given to regions that have only one or two aberrant CN states and a high density of CN state oscillations. For example, a region with 3 different aberrant CN states, each contributing 5 CN state oscillations to the region, would receive a lower CNV score than a region with only 1 aberrant CN state that contributed 15 CN state oscillations. To achieve this sensitivity, we calculate:

(4) $$\;\text{CNV}\;\text{Score}=\left(\frac{1}{N_{\text{significant}}}\right)\;\;\left(1-\frac{1}{1+{\text{log}}_{2}(\frac{\sum_{n=1}^{N_{\text{significant}}}c_{n}^{2}}{N_{\text{significant}}})}\right)$$

*N*_*significant*_ is determined from the standard deviation of the number of regions at each aberrant CN level. The z-score for each value is then calculated and *N*_*significant*_ is set to the number of values that have a z-score greater than -2 (this is user definable). This is done to filter noisy calls that incorrectly add CN states to the region. As such, the first term in the equation reduces the aberrant CN score as the number of CN states that contribute a significant number of events increases. The second term increases the aberrant CN score as the number of region contributed by significant CN states increases and gives more power to CN state oscillations contributed by a single CN state versus those contributed by many CN states.

#### Translocation localization

The equations that are used to calculate this hallmark score aim to give a high score to regions that have a high number of localized translocations and give a low score to regions with only a few translocations that are not localized. For each highly mutated region, chromosomes involved in a translocation with the region are identified and, for each of these, a list of translocation breakpoints is generated. For example, if in a highly mutated region found in chromosome 3 there exists an inserted fragment that matches a sequence in chromosome 1, then the breakpoints on chromosome 1 that delimit the translocated fragment will be recorded. Additionally, if in the same highly mutated region there exists a sequence that matches an inserted fragment on chromosome 2, then the breakpoints on chromosome 2 that delimited this fragment will be recorded. The translocation score is then:

(5) $$\;\text{spread}=\frac{\sum_{i=1}^{C}b_{i+1}-b_{i}}{C}$$

(6) $$\;\text{spread factor}=\frac{\text{log}(1+{\text{spread}}_{n})}{-\text{log}(\text{expected}\;\text{spread})}$$

(7) $$\;\text{weighted sum}=\overset{N_{\text{sig}}}{\underset{n=1}{\sum }}\left(\frac{c_{n}^{2}}{\left(\text{spread}\;\text{factor})(c_{T}\right)}\right)$$

(8) $$\begin{array}{l} \text{Translocation Score}=\left(1-0.10(N_{\text{significant}}-2)\right)\\ \;\;\times \left(1-\frac{1}{{\text{log}}_{2}(1+\text{weighted}\;\text{sum})}\right) \end{array}$$

The value of *N*_*significant*_ in equation (8) indicates the number of chromosomes that share a significant number of translocation with the highly mutated region. *N*_*significant*_ is determined by calculating the standard deviation of the number of translocations between each chromosome and the region, and then calculating the z-score of each value. *N*_*significant*_ is the number of z-scores that are greater than a user definable value. By default this value is -2, which serves to remove the chromosomes with translocation counts in the bottom 3% of all the counts. This prevents low-value outliers from over influencing the translocation score. If *N*_*significant*_ is greater than 8 (a user-definable parameter) then the value of the first term in equation (8) and thus the entire translocation score is set to 0. The default cut-off value of 8 reflects our observation that most documented cases of chromothripsis only had translocation between a small number of chromosomes. If the value of *N*_*significant*_ is 1 then the value of the first term is set to 1.

The second term in equation (8) increases the score as the total number of translocations to or from the highly mutated region increases. The *weighted sum* is a scaled sum of the translocation counts of each significant chromosome. This value is calculated in equation (7). *c*_n_ is the number of translocations from significant chromosome *n* and *c*_T_ is the total number of translocations from all significant chromosomes. Thus the translocation count for each chromosome is scaled by the fraction of the total number of translocations that are contributed by that chromosome. Additionally, the count from each chromosome is scaled according to the spread of the translocation breakpoints along that chromosome. The larger the spread, the more the translocation count is reduced and the smaller the spread the greater the count is amplified. To determine the *spread factor*, the calculated *spread* is compared to the *expected spread* in equation (6).

The *spread* for each chromosome is determined by first calculating the difference between adjacent terms in an ordered list of the breakpoints on the chromosome. These breakpoints correspond only to translocations that are to or from the highly mutated region. The standard deviation of the separation values is then determined and values that are found to have a z-score greater than 2 (this is a user definable value) are disregarded. High-value outliers are removed from the calculation in order to not penalize cases where there are multiple small groups of localized translocation breakpoints, each separated by some relatively large distance. The *spread* is then calculated in equation (5) as the mean of the remaining separation values. The combination of these equations gives a high score to regions with very localized translocations between one or two chromosomes.

#### Regions of retained heterozygosity

To calculate the heterozygosity score for a highly mutated region, ShatterProof first makes an estimate about how much heterozygosity should remain in the mutated chromosome. ShatterProof assumes that all the regions between any CNV are heterozygous, then using any experimental LOH data, it calculates the percentage of predicted heterozygous regions that overlap with regions of LOH. The heterozygosity score is:

(9) $$\;\begin{array}{c} \text{Heterozygosity}\;\text{Score}=1-\frac{\text{Amount}\;\text{of}\;\text{LOH}\;\text{Overlap}}{\text{Amount}\;\text{of}\;\text{Original}\;\text{Heterozygosity}} \end{array}$$

High LOH overlap indicates a large loss of heterozygosity, resulting in a low score. Limited overlap indicates that most heterozygosity between CNVs is preserved, resulting in a high score.

#### Short insertions at breakpoints

The breakpoint insertion score is calculated by determining the fraction of translocation breakpoints in a highly mutated region where short insertions are found within 10 base pairs of the breakpoint.

(10) $$\;\text{Short}\;\text{Insertion}\;\text{Score}=\frac{\text{Number}\;\text{Of}\;\text{Breakpoints}\;\text{With}\;\text{Insertions}}{\text{Total}\;\text{Number}\;\text{Of}\;\text{Breakpoints}}$$

The greater the fraction, the higher the score.

#### TP53 mutations

The TP53 mutation score is simply a 1 or 0 depending on whether or not the user indicates that a non-synonymous mutation is present in the TP53 loci. This feature can be used if TP53 mutations are identified via non-sequencing techniques(*e.g.* Immunohistochemistry). Additionally, ShatterProof will scan input data and determine if any mutations are present in the 7.57 -7.59 Mbp region of chromosome 17. If mutations are found, ShatterProof will note in the final output file that TP53 mutations were discovered, however these mutations will not affect the chromothripsis scores.

#### Output

ShatterProof produces a number of different output files which summarize the analysis that it performs. The output files can be divided into three categories: final report, intermediate data, and TP53 mutations.

The final report file lists the regions where ShatterProof has found strong indications of chromothripsis. For each suspect region, the output file contains the chromosome, start and end position of the region, the chromothripsis score, and additional statistics about each of the chromothriptic hallmarks. YAML was chosen as the output format so that it would be both human readable as well as easily machine parseable. A sample final report file is shown in Appendix B (Additional file 3).

The intermediate data output files contain: 

•the results of the sliding window analysis of the SV clustering

•the number of CN state oscillations on each chromosome

•the overall SV density of each chromosome

•a list of the number of aberrant CN states present on each chromosome

•a list of the number of translocations that occur between each and every chromosome

**Figure 3:**
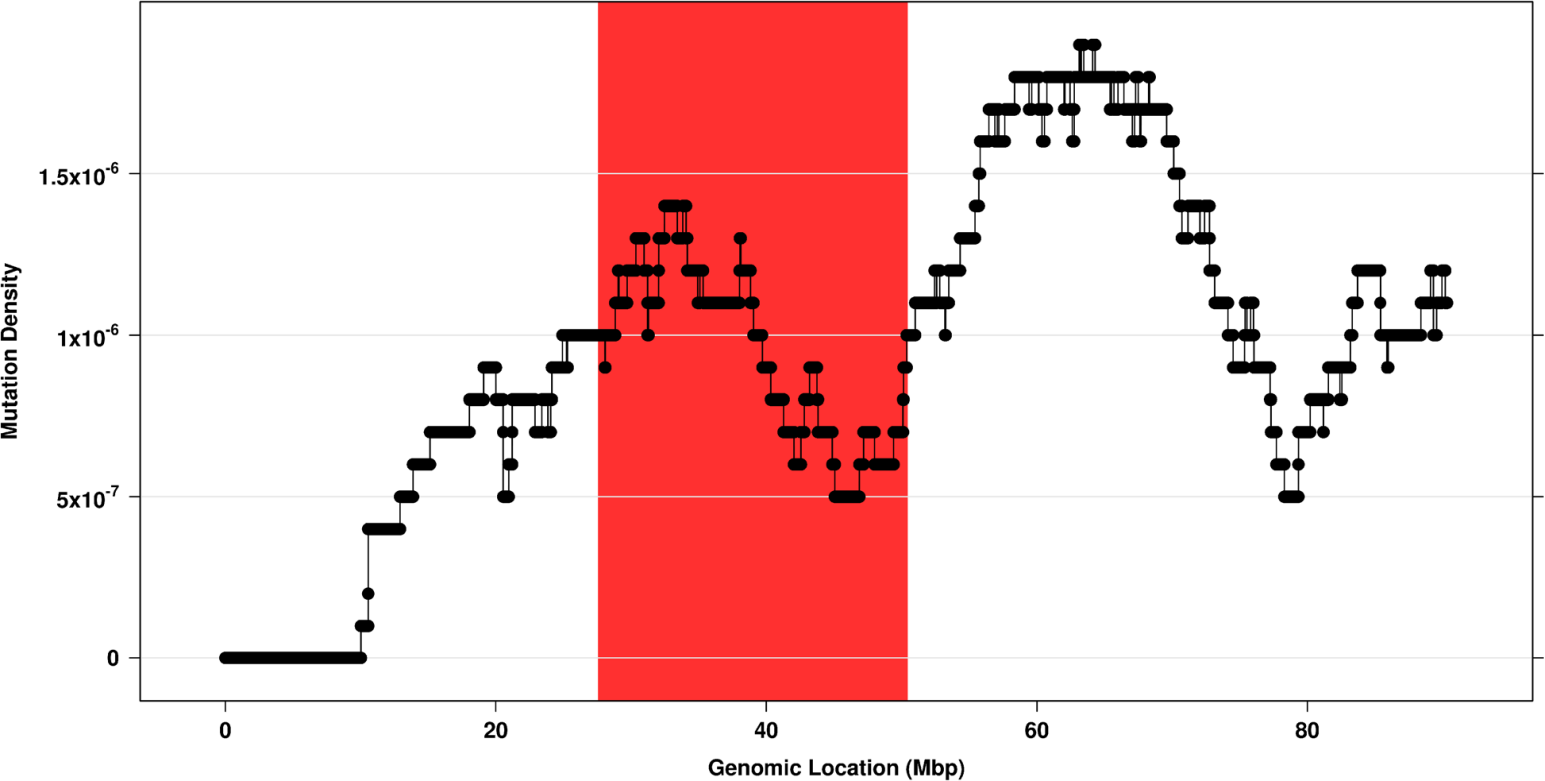
**Sliding window analysis.** Graphing the data from the tab delimited intermediate output files produces plots that visualize the clustering of SVs along a chromosome. The region highlighted in red was identified by ShatterProof as highly chromothriptic. Note that chromothriptic regions are not always those with the highest SV density. ShatterProof identifies regions as chromothriptic primarily based on the organization and clustering of SVs.

Intermediate data output files are tab delimited so that they can easily be graphed using software such as R or MATLAB. For example, the data from the sliding window analysis of a sample chromosome was visualized using R (Figure 3).

## Results

### Validation

To validate the accuracy of ShatterProof, we ran the tool against 21 separate whole-genome sequences (Table 2).

**Table 2:** Summary of clinical data

**Sample type**	**Source**	**Count**	**Coverage**	**Reference**
Normal blood	CPCG network	10	30 ×	hg19
Prostate cancer	CPCG network	7	50 ×	hg19
Prostate cancer	Living tumor lab	2	3 ×	hg18
Colorectal cancer	[1]	1	50 ×	hg19
SCLC	[1]	1	50 ×	hg19

All sequences were aligned using Novoalign (Novocraft v2.07.14). Translocation calls were produced using BreakDancer (v1.1) [17]. Only translocation calls that had a confidence score greater than 60 were considered. When running both the pre-processing and main phase of BreakDancer, no command line options were supplied. Control-FREEC (v5.7) [18] was used to produce CNV calls for all of the samples. See Additional file 4 for the configuration file used to run Control-FREEC.

We first ran ShatterProof on 10 normal reference samples. ShatterProof produced an average of 10 calls (9 - 12) per sample with a median score of 0.1468 (0.1089 - 0.3667). The low number of calls produced and their low scores are consistent with the fact that these samples contain few SVs that are not highly localized. This provides a background distribution for ShatterProof.

Next, we ran ShatterProof against data from the 2 samples from the Living Tumor Lab (Accession Number: [SRX147666] and [SRX147668]). Four replicate libraries were provided for each sample, previously described by Wu *et al.*[19]. The samples were found to bear both the translocation and CNV hallmarks of chromothripsis on chromosomes 4, 8, 12, and 20, with translocation breakpoints clustered very closely to the CNV breakpoints resulting in a few localized regions of very high SV density on those chromosomes. Wu *et al.* utilized data from both RNA and DNA sequencing to produce accurate SV calls which they then further analyzed to call chromothripsis [19].

To produce the translocation and CNV calls that were given to ShatterProof, we provided BreakDancer and Control-FREEC with DNA sequencing data from each of the 8 replicate libraries. Running ShatterProof with these SV calls produced an average of 16 chromothriptic calls (11 - 21) per replicate with a median score of 0.2797 (0.159 - 0.558). The regions which were scored highest by ShatterProof, with scores ranging from 0.43 to 0.558, correspond to those previously identified as highly chromothriptic [19]. Specifically, ShatterProof produced 17 calls with scores greater than 0.43, all of which came from chromosomes 4, 8, 12, or 20. These results demonstrate ShatterProof’s ability to accurately call chromothripsis from DNA sequencing data alone. See Additional files 5 and 6 for diagrams of translocation and CNV calls.

We also acquired the colorectal adenocarcinoma and SCLC data sets used in the first description of chromothripsis [1] (Accession Number: [EGAD00001000002]) and performed our own analysis on these samples. [1] identified chromothripsis as having occurred on chromosome 15 of the colorectal adenocarcinoma sample and on chromosome 8 of the SCLC sample. Providing ShatterProof with translocation and CNV calls from the colorectal adenocarcinoma sample produced 5 chromothriptic calls (4 from chromosome 15) with a median score of 0.4166 (0.174 - 0.450). See Additional file 7 for a diagram of translocation and CNV calls. Similarly, when ShatterProof was used to analyze the SV calls from the SCLC sample, 17 chromothriptic calls were produced (14 from chromosome 8) with a median score of 0.3196 (0.207 - 0.494). See Additional file 8 for a diagram of translocation and CNV calls. In both cases, the highest scoring calls identified the regions of these genomes that were described as bearing hallmarks strongly indicative of chromothripsis [1].

Additionally, we ran ShatterProof against 7 primary prostate adenocarcinoma samples, which have not previously been described. ShatterProof produced an average of 11 (9 - 13) calls per sample with a median score of 0.1437 (0.101 - 0.337). While these samples had a higher total number of SVs than samples from other sets, a lack of clustering of translocations and CNVs resulted in low chromothripsis scores. To ensure the robustness of these results, we validated our predictions using PCRbased analysis of the breakpoints (Additional file 9). We also verified that similar score-distributions and conclusions were obtained using diverse SV-callers (data not shown), providing confidence that our conclusions are independent of the SV-calling algorithm.

**Figure 4:**
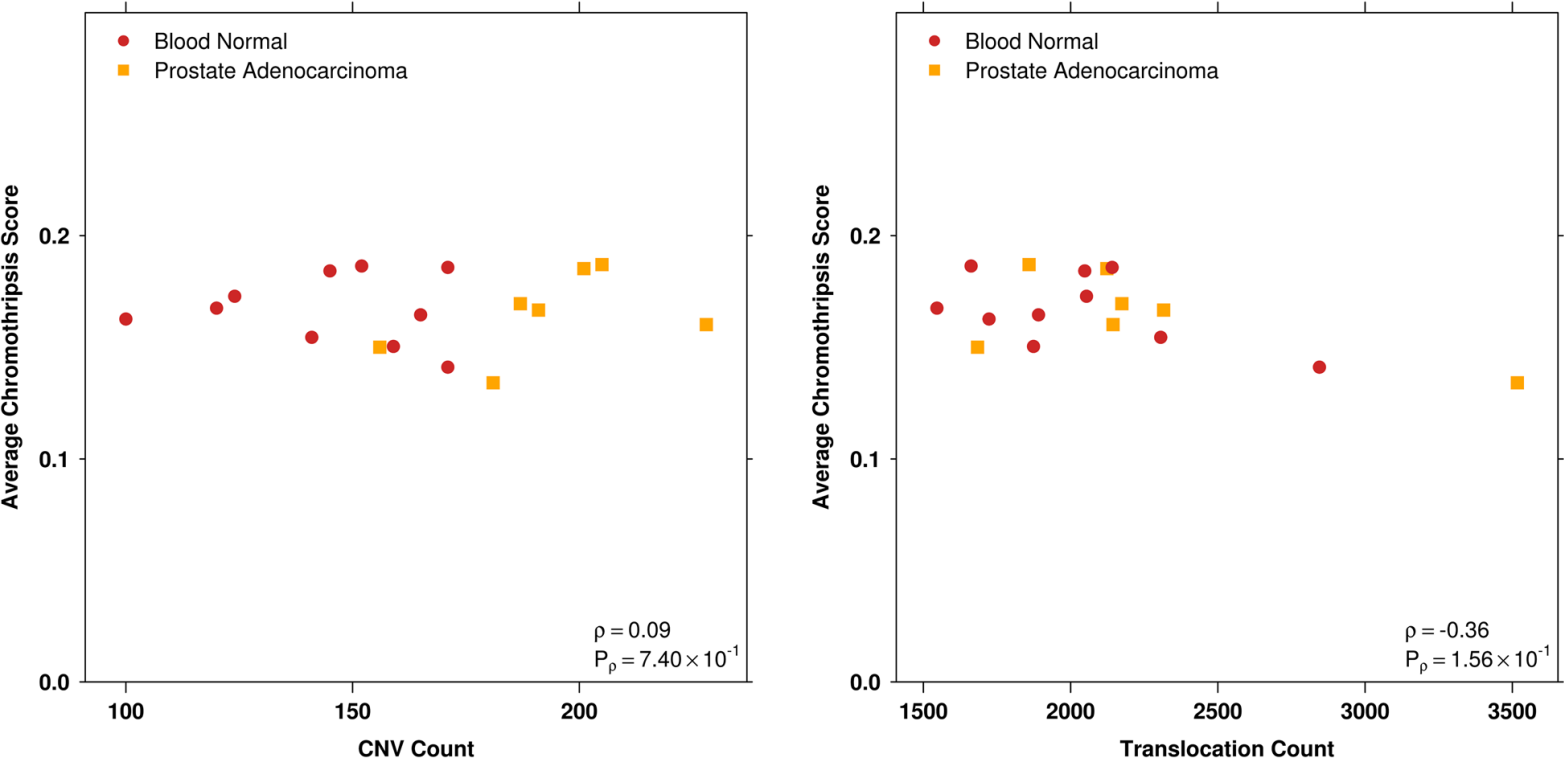
**CNV and translocation count vs average chromothripsis score.** These plots illustrates how a high total SV count does not necessarily produce high chromothripsis scores. Samples producing the highest chromothripsis scores had some of the lowest translocation and CNV counts, showing that ShatterProof scores are dominated by the clustering of SVs, not the absolute counts.

The results from the analysis of the prostate adenocarcinoma samples that had not previously been described, demonstrates the important fact that ShatterProof produces scores which primarily reflect the organization and clustering of SVs as opposed to simply the absolute counts of translocations and CNVs. Figure 4 illustrates how the chromothripsis scores that ShatterProof produces are in fact uncorrelated with the absolute counts of translocations and CNVs.

**Figure 5:**
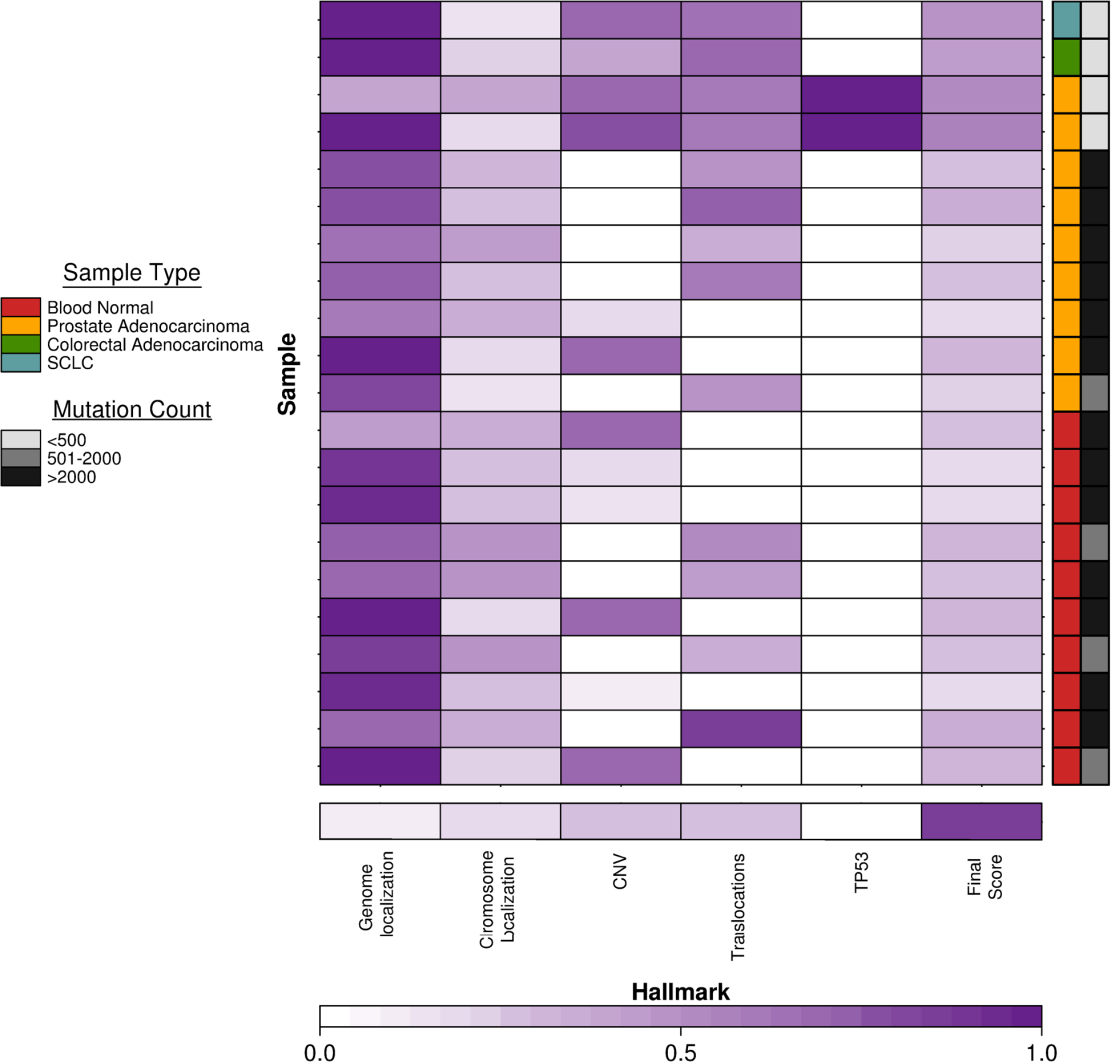
**Hallmark scores of highest scoring region per sample.** The bottom row indicates the relative weightings assigned to each hallmark via MCA and thus the maximum possible score that can be achieved. All rows above the bottom one depict the contribution of each hallmark score to the highest final score for a sample. Dark purple indicates high scores; white indicates low scores. The two samples expressing the TP53 hallmark correspond to the highest scores from the 2 LTL samples.

We see in Figure 4 that ShatterProof scores are not simply a measure of the number of SVs in a sample. Rather, the scores produced by ShatterProof reflect the extent to which a sample exhibits the hallmarks of chromothripsis. Figure 4 also demonstrates the fact that many cancer samples with very high rates of genomic instability will produce low scores that are similar to those of normal samples if chromothriptic hallmarks are not discovered. Figure 5 illustrates how the hallmark scores for the highest scoring region in each sample contributed to the final score for that region.

**Figure 6:**
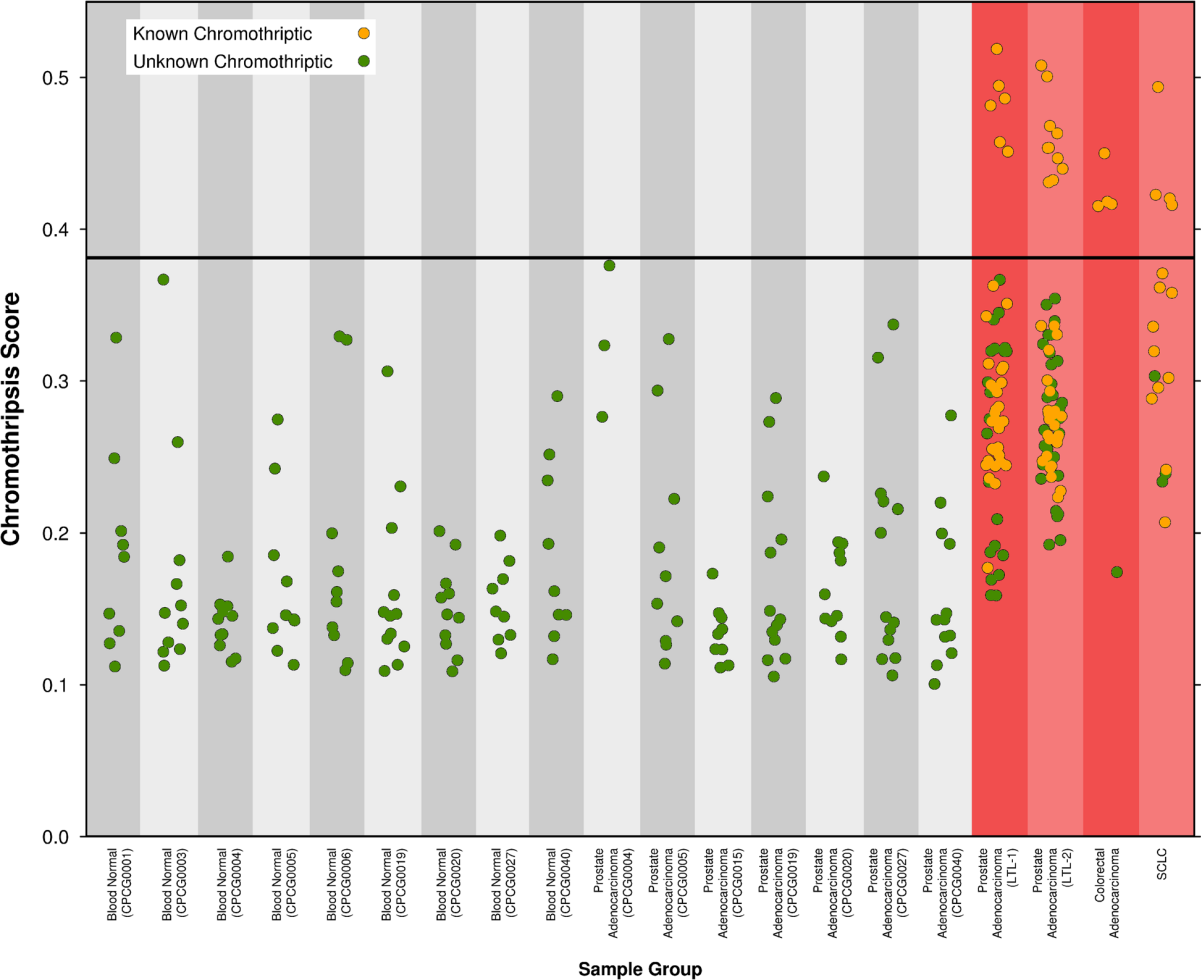
**Final chromothripsis scores.** All scores produced by ShatterProof for each sample. The gold symbols represent calls made by ShatterProof that correspond to regions of the genomes that were previously identified in other studies as being chromothriptic. Samples highlighted in red produced numerous calls with scores over 0.37 and as such are believed to have experienced a chromothriptic event.

We found that the calls ShatterProof produced for the blood normal samples had a maximum value of 0.3667. Figure 6 illustrates how the samples that were known to be chromothripsis positive (prostate adenocarcinoma samples from the LTL, colorectal adenocarcinoma samples, and the SCLC samples) all produced multiple calls with scores that were greater than 0.37. Furthermore, it is the case that all of these high scoring calls correctly identified regions that had been identified in other studies as being chromothriptic.

Based on these two findings, we suggest that when running ShatterProof with the default hallmark weightings, a call with a score that exceed 0.37 identifies a region of the genome in which it is likely that chromothripsis has occurred. Using this cut-off we analyzed the results that ShatterProof produced for the 7 prostate adenocarcinoma samples which had not been previously analyzed for signs of chromothripsis. We found that 0 calls with a score that exceeded 0.37 were produced. This strongly suggests that none of these tumor samples have experienced any chromothriptic events.

### Performance

ShatterProof performance is most dependent on the genome bin size parameter. This parameter defines the size of bins into wich the genome is divided. A smaller bin size will require ShatterProof to consume more memory and cause the sliding window analysis to take longer. Specifically, memory consumption and runtime are inversely realted to bin size (*i.e.* O(1/n)). Additional file 10 illustrates this relationship. Changing the window size or number of input lines (representing CNV, translocation, and LOH calls) has a limited effect on performance relative to changing the bin size. However, in general, increasing the window size decreases the time required to perform the sliding window analysis and increasing the number of input lines increases run time as well as memory consumption. Testing was performed using a single core (Intel(R) Xeon(R) CPU E5540 @ 2.53 GHz) with 8 GB of physical memory. When ShatterProof was run using the default config file supplied with the ShatterProof distribution (Bin Size: 1000, Window Size: 10000), it consumed 670 MB of memory and its run time was on average 60 seconds with a standard deviation of 3.5 seconds across 10 trial runs.

## Discussion

Use of ShatterProof will mitigate the shortcomings of *ad hoc* characterization by generating a wide range of detailed metrics which allow for more accurate identification and precise quantification of chromothriptic events. ShatterProof relies on SV calls that are produced by other tools and as such its output is dependent on the tools chosen as well as their parameterization. Consequently, ShatterProof scores are not directly comparable if different tools were used to produce input data. Additionally, if the hallmark weightings used to produce scores are different between projects, then direct score comparisons will be ambiguous. However, ShatterProof provides a standardized pipeline that allows researchers to quickly identify chromothripsis in large amounts of data and produce detailed metrics which can be easily reproduced by others.

The definition of chromothripsis, including what exactly are its hallmarks, remains controversial. At time of writing there is no singular and clear definition of chromothripsis, and as such ShatterProof must rely on heuristics in order to identify chromothriptic candidates. Realizing that the definition of chromothripsis may change over time, we designed ShatterProof to use many user-definable parameters so that as the definition of chromothripsis evolves, the tool could be easily adapted to continue to provide accurate analysis of data.

Indeed, we hope the data produced by ShatterProof will help lead to a more detailed and concrete characterization of chromothripsis by giving a common and extensible language for describing this phenomenon. Future development work planned for ShatterProof includes augmenting the scoring equations to consider the quality values of SV calls when calculating hallmark scores. Additionally, we are investigating using machine learning to improve the precision of identification. As sequencing of additional chromothriptic tumours happens, training sets can be created and used to obviate the need for hallmark weightings and lead to a better characterization of chromothriptic events.

## Conclusion

The discovery of chromothripsis has revealed a new mechanism by which cancer genomes evolve. The wide variety of cancers in which chromothripsis occurs speaks to the importance of further investigation. ShatterProof accurately and efficiently annotates occurrences of chromothripsis in genomic data and can easily be integrated into existing sequence analysis pipelines. Use of ShatterProof will enable researchers to quickly screen a large number of samples and thus improve the detection rate of chromothripsis. As the prevalence of whole-genome sequencing increases, the need for tools such as ShatterProof which allow the rapid and accurate analysis of large-scale data will continue to increase.

## Availability and requirements

**Project name:** ShatterProof

**Project home page:**http://search.cpan.org/~sgovind/Shatterproof

**Operating system(s):** Platform independent

**Programming language:** Perl

**Other requirements:** Perl version>5.10

**License:** GNU GPL v3.0

**Any restrictions to use by non-academics:** None

## Abbreviations

SV: Structural variation; CNV: Copy number variation; VCF: Variant call format; CN: Copy number; LOH: Loss of heterozygosity.

## Competing interests

The authors declare that they have no competing interests.

## Authors’ contributions

The project was initiated by PCB and SKG. The analysis algorithm and software were designed and implemented by SKG with assistance from AZ. Sample tumor data was provided by MF, TvdK, AWW, CCC and by RGB. Input data was produced by SKG, AZ, and PHH. Data visualization was performed by SKG and CA. ShatterProof was run and the results were analyzed by S.K.G. The initial paper was written and figures were produced by SKG. JDW performed PCR validation of a subset of the translocation calls. All authors approved final draft of the paper. PCB, RGB, CCC, and JDM provided project supervision. All authors read and approved the final manuscript.

## Supplementary Material

Additional file 1 **Supplementary Figure 1 - example input file formats.** Examples of the three types of input file formats that ShatterProof reads. Click here for file

Additional file 2 **MCA calculation matrix.** Spreadsheet document used to calculate hallmark weightings. Click here for file

Additional file 3 **Appendix.** The Appendix includes a more detailed description of the MCA process as well as a sample final report file. Click here for file

Additional file 4 **FREEC configuration file.** Configuration file that was used to run Control-FREEC. Click here for file

Additional file 5 **Supplementary Figure 2 - Clustering of SVs to chromosome 4,8,12, and 20 of prostate adenocarcinoma genome (LTL-1).** Circos plot of prostate cancer adenocarcinoma genome (sample LTL-1). From outermost ring going inward each ring indicates: cytogenetic bands, genetic density, histogram of CNV locations, and link diagram of translocation data (interchromosomal in blue, intrachromosomal in red). The plot demonstrates clustering of structural variation to chromosomes 4,8,12, and 20. Click here for file

Additional file 6 **Supplementary Figure 3 - Clustering of SVs to chromosome 4,8,12, and 20 of prostate adenocarcinoma genome (LTL-2).** Circos plot of prostate cancer adenocarcinoma genome (sample LTL-2). From outermost ring going inward each ring indicates: cytogenetic bands, genetic density, histogram of CNV locations, and link diagram of translocation data (interchromosomal in blue, intrachromosomal in red). The plot demonstrates clustering of structural variation to chromosomes 4,8,12, and 20. Click here for file

Additional file 7 **Supplementary Figure 4 - Clustering of SVs to chromosome 15 of colorectal cancer genome.** Circos plot of colorectal cancer genome. From outermost ring going inward each ring indicates: cytogenetic bands, genetic density, histogram of CNV locations, and link diagram of translocation data (interchromosomal in blue, intrachromosomal in red). The plot clearly demonstrates a clustering of structural variation to chromosome 15. Click here for file

Additional file 8 **Supplementary Figure 5 - Clustering of SVs to chromosome 8 of SCLC genome.** Circos plot of SCLC genome. From outermost ring going inward each ring indicates: cytogenetic bands, genetic density, histogram of CNV locations, and link diagram of translocation data (interchromosomal in blue, intrachromosomal in red). The plot clearly demonstrates a clustering of structural variation to chromosome 8. Click here for file

Additional file 9 **PCR validation methods and data.** This document contains the methods used to perform PCR validation on a subset of the translocation calls presented in the paper as well as the specific primer sequences and the validation results. Click here for file

Additional file 10 **Supplementary Figure 6 - Run time and memory consumption.** These plots illustrate the inverse relationship between run time/memory consumption and bin size. The error bars on the run time vs bin size plot indicate the range of times that were observed across 10 trial runs. No error bars are present on the memory consumption vs bin size plot as we found memory consumption to be consistent between trial runs using the same bin size. Click here for file

## References

[B1] Stephens PJ Greenman CD Fu B Yang F Bignell GR Mudie LJ Pleasance ED Lau KW Beare D Stebbings LA McLaren S Lin M McBride DJ Varela I Nik-Zainal S Leroy C Jia M Menzies A Butler AP Teague JW Quail MA Burton J Swerdlow H Carter NP Morsberger LA Iacobuzio-Donahue C Follows GA Green AR Flanagan AM Stratton MR Massive genomic rearrangement acquired in a single catastrophic event during cancer development Cell 2010 144 27 40 21215367 10.1016/j.cell.2010.11.055 PMC3065307

[B2] Crasta K Ganem NJ Dagher R Lantermann AB Ivanova EV Pan Y Nezi L Protopopov A Chowdhury D Pellman D DNA breaks and chromosome pulverization from errors in mitosis Nature 2012 482 7383 53 58 10.1038/nature10802 22258507 PMC3271137

[B3] Rausch T Jones DT Zapatka M Stutz AM Zichner T Weischenfeldt J Jager N Remke M Shih D Northcott PA Pfaff E Tica J Wang Q Massimi L Witt H Bender S Pleier S Cin H Hawkins C Beck C von Deimling A Hans V Brors B Eils R Scheurlen W Blake J Benes V Kulozik AE Witt O Martin D Genome sequencing of pediatric medulloblastoma links catastrophic, DNA rearrangements with TP53 mutations Cell 2012 148 59 71 10.1016/j.cell.2011.12.013 22265402 PMC3332216

[B4] Kloosterman W Hoogstraat M Paling O Tavakoli-Yaraki M Renkens I Vermaat J van Roosmalen M van Lieshout S Nijman I Roessingh W van ’t Slot R van de Belt J Guryev V Koudijs M Voest E Cuppen E Chromothripsis is a common mechanism driving genomic rearrangements in primary and metastatic colorectal cancer Genome Biol 2011 12 10 R103 10.1186/gb-2011-12-10-r103 22014273 PMC3333773

[B5] Zhang F Carvalho CM Lupski JR Complex human chromosomal and genomic rearrangements Trends Genet 2009 25 7 298 307 10.1016/j.tig.2009.05.005 19560228 PMC4464790

[B6] Zhang CZ Mitchel LL Pellman D Chromothripsis and beyond: rapid genome evolution from complex chromosomal rearrangements Genes Dev 2013 27 2513 2530 10.1101/gad.229559.113 24298051 PMC3861665

[B7] Liu P Erez A Nagamani SCS Dhar SU KoBodziejska KE Dharmadhikari AV Cooper ML Wiszniewska J Zhang F Withers MA Bacino CA Campos-Acevedo LD Delgado MR Freedenberg D Garnica A Grebe TA Hernandez-Almaguer D Immken L Lalani SR McLean SD Northrup H Scaglia F Strathearn L Trapane P Kang SL Patel A Cheung SW Hastings PJ Stankiewicz P Lupski JR Chromosome catastrophes involve replication mechanisms generating complex genomic rearrangements Cell 2011 146 6 889 903 10.1016/j.cell.2011.07.042 21925314 PMC3242451

[B8] Kim TM Xi R Luquette LJ Park RW Johnson MD Park PJ Functional genomic analysis of chromosomal aberrations in a compendium of 8000 cancer genomes Genome Res 2013 23 2 217 227 10.1101/gr.140301.112 23132910 PMC3561863

[B9] Northcott PA Shih DJH Peacock J Garzia L Morrissy AS Zichner T Stutz AM Korshunov A Reimand J Schumacher SE Beroukhim R Ellison DW Marshall CR Lionel AC Mack S Dubuc A Yao Y Ramaswamy V Luu B Rolider A Cavalli FMG Wang X Remke M Wu X Chiu RYB Chu A Chuah E Corbett RD Hoad GR Jackman SD Subgroup-specific structural variation across 1,000 medulloblastoma genomes Nature 2012 488 7409 49 56 10.1038/nature11327 22832581 PMC3683624

[B10] Baca SC Prandi D Lawrence MS Mosquera JM Romanel A Drier Y Park K Kitabayashi N MacDonald TY Ghandi M Allen EV Kryukov GV Sboner A Theurillat JP Soong TD Nickerson E Auclair D Tewari A Beltran H Onofrio RC Boysen G Guiducci C Barbieri CE Cibulskis K Sivachenko A Carter SL Saksena G Voet D Ramos AH Winckler W Punctated evolution of prostate cancer genomes Cell 2013 153 3 666 677 10.1016/j.cell.2013.03.021 23622249 PMC3690918

[B11] Molenaar JJ Koster J Zwijnenburg DA van Sluis P Valentijn LJ van der Ploeg I Hamdi M van Nes J Westerman BA van Arkel J Ebus ME Haneveld F Lakeman A Schild L Molenaar P Stroeken P van Noesel MM Ora I Santo EE Caron HN Westerhout EM Versteeg R Sequencing of neuroblastoma identifies chromothripsis and defects in neuritogenesis genes Nature 2012 483 7391 589 593 10.1038/nature10910 22367537

[B12] Holland AJ Cleveland DW Chromoanagenesis and cancer: mechanisms and consequences of localized, complex chromosomal rearrangements Nature Med 2012 18 11 1630 1638 10.1038/nm.2988 23135524 PMC3616639

[B13] Kloosterman WP Tavakoli-Yaraki M Roosmalen MJ Binsbergen E Renkens I Duran K Ballarati L Vergult S Giardino D Hansson K Ruivenkamp CAL Jager M Haeringen A Ippel EF Haaf T Passarge E Hochstenbach R Menten B Larizza L Guryev V Poot M Cuppen E Constitutional chromothripsis rearrangements involve clustered double-stranded DNA breaks and nonhomologous repair mechanisms Cell Rep 2012 1 6 648 655 10.1016/j.celrep.2012.05.009 22813740

[B14] YAML ain’t Markup Language (YAML) Version 1.2 [http://www.yaml.org/spec/1.2/spec.html]

[B15] VCF (Variant Call Format) version 4.0 [http://www.1000genomes.org/node/101]

[B16] The analytic hierarchy process [http://msdn.microsoft.com/en-us/magazine/cc163785.aspx]

[B17] Chen K Wallis JW McLellan MD Larson DE Kalicki JM Pohl CS McGrath SD Wendl MC Zhang Q Locke DP Shi X Fulton RS Ley TJ Wilson RK Ding L Mardis ER BreakDancer: an algorithm for high-resolution mapping of genomic structural variation Nat Methods 2009 6 9 677 681 10.1038/nmeth.1363 19668202 PMC3661775

[B18] Boeva V Zinovyev A Bleakley K Vert JP Janoueix-Lerosey I Delattre O Barillot E Control-free calling of copy number alterations in deep-sequencing data using GC-content normalization Bioinformatics 2011 27 2 268 269 10.1093/bioinformatics/btq635 21081509 PMC3018818

[B19] Wu C Wyatt A McPherson A Lin D McConeghy B Mo F Shukin R Lapuk A Jones S Zhao Y Marra M Gleave M Volik S Wang Y Sahinalp S Collins C Ploy-Gene Fusion Transcripts and Chromothripsis in Prostate Cancer Genes Chromosomes Cancer 2012 51 12 1144 1153 10.1002/gcc.21999 22927308

