# Supplementary material for: ShatterProof: operational detection and quantification of chromothripsis
Source: BMC Bioinformatics. 2014 Mar 19;15:78. doi: 10.1186/1471-2105-15-78 (PMC3999944; doi:10.1186/1471-2105-15-78)
Supplement: Additional file 1 — Supplementary Figure 1 - example input file formats. Examples of the three types of input file formats that ShatterProof reads. [file 1471-2105-15-78-S1.pdf]

Translocation Input File Format

```
#chr1   start   end     chr2    start   end     quality
chr1    13173372        13178884        chr1    13182123        13187635        0.94
chr15   20015951        20016127        chr21   14362848        14363024        0.63
```

Copy Number Variation Input File Format

```
#chr    start   end     number  quality
chr10   38780000        38818999        1       0.72
chr9    130893000       130900999       4       0.41
```

Loss of Heterozygosity Input File Format

```
#chr    start   end     quality
chr2    171788652       173965180       0.53
chr4    14029390        14958821        0.99
```
