# Supplementary material for: ShatterProof: operational detection and quantification of chromothripsis
Source: BMC Bioinformatics. 2014 Mar 19;15:78. doi: 10.1186/1471-2105-15-78 (PMC3999944; doi:10.1186/1471-2105-15-78)
Supplement: Additional file 3 — Appendix. The Appendix includes a more detailed description of the MCA process as well as a sample final report file. [file 1471-2105-15-78-S3.pdf]

Appendix A

The first step in the MCA after having decided upon criteria, is to establish a scale of relative importance. This is used to compare all of the criterion to each other and establish their relative importance. The scale used in our analysis is given in Table A1:

Supplementary Table A1 Rating Scale

**Relative Importance**	
Equally Important	1
Slightly More Important	2
Significantly More Important	3
Much More Important	4
Extremely More Important	5

Once the scale has been decided on, a matrix is used to assigns the numerical values for relative importance to each criterion. The left most column and the top most row contain the criteria. The values placed in the remaining cells indicate the relative importance of the criterion in that row, to the criterion in that column.

The matrix of the chromothriptic hallmarks is presented in Table A2 in the Supplementary Tables file.

The cells of the matrix that are below the diagonal were filled in with the scaled importance value of each criterion. The matrix has the property that:

$$cell_{ij} = cell_{ji}^{-1} \tag{1}$$

As such the values in the cells above the diagonal are auto generated from the user defined values of the cells below the diagonal.

Justification for Relative Importances

The clustering of mutations to a specific region of a chromosome was considered to be significantly more important that than the clustering of mutations to a specific chromosome relative to the entire genome for a number of reasons. Observed cases of chromothripsis have always included the occurrence of a large number of mutations localized to a small area of one or two chromosomes. While it has also been the case that the mutations found in these clusters made up a large fraction of the mutations found in the entire genome, it is also reasonable to predict that a large number of chromosomes could each have highly mutated regions. A genome that contained such chromosomes would exhibit reduced clustering of mutations with respect to the whole genome but possibly still have experienced a chromothriptic event. As such, the localization of mutations to a

specific region of a chromosome was considered to be a significantly more important hallmark of chromothripsis than the localization of mutations to a specific chromosome within the genome.
The copy number variation hallmark was considered to be much more important than the genome localization hallmark. This hallmark is somewhat unique to chromothripsis and as such the presence of this hallmark is a very strong indicator that chromothripsis has occurred. We would sooner predict the occurrence of chromothripsis in a sample that had limited localization of mutations in the genome but displayed the distinctive copy number profile in some locations then in a sample that had localization of mutations but no distinctive pattern of copy number variations.
The translocation hallmark was also considered to be much more important than the genome localization hallmark for reasons similar to the CNV hallmark.
The retention of heterozygosity hallmark was considered to be significantly less important than the genome localization hallmark since its absences would not preclude calling the occurrence of chromothripsis in a sample. Whereas if the genome localization hallmark were not expressed in a sample, it is unlikely that chromothripsis would be called in that sample.
The presences of short insertions at translocations breakpoints was considered to be significantly less important than the genome localization hallmark for reasons similar to the retention of heterozygosity hallmark. Additionally, the presences of translocations is highly indicative of the shattering of a chromosome which has been identified in all cases, whereas short insertions are indicative of only one of the proposed stitching processes which has not been observed in all cases. As such, it would be very reasonable to call the occurrence of chromothripsis in samples that exhibit mutation clustering but lack short insertions at translocation breakpoints.
The TP53 mutation hallmark was considered to be much less important than the genome localization hallmark since this hallmark has only been discovered in a subset of cases, and due to the fact the presence of this hallmark alone, would be an extremely weak marker for chromothripsis. **Weighting Calculation**

The next step in the process is to normalize the values in the importance matrix. This is done by dividing each value by the sum of all the values in the same column. The normalized matrix for the hallmark weightings is given in Table A3 in the Supplementary Tables file.
The final step in calculating the relative weightings for the hallmarks is to calculate the average of each row of the normalized matrix. These values are the quantitative weightings that will be assigned to the hallmark for that row. The final rounded weighting for each hallmark is given in Table A4 below:

Supplementary Table A4 Final Hallmark Weights

**Hallmark**	**Weight**
Genome Localization	0.1145
Chromosome Localization	0.1697
CNVs	0.2724
Translocations	0.2724
Retention of Heterozygosity	0.0648
Short Insertions	0.0657
TP53	0.0406

Appendix B

Sample final report file:

```
file: Suspect Chromothriptic Regions
bin_size:                          1000
localization_window_size:          10000

genome_localization_score_weight:          0.1145
chromosome_localization_score_weight:      0.1697
cnv_score_weight:                          0.2724
translocation_score_weight:                0.2724
insertion_breakpoint_score_weight:         0.0657
loh_score_weight:                          0.0648
tp53_mutation_score_weight:                0.0406

min_mutation_density_z_score:      2
---

chromosome:        4
start:             181025000
end:               191264000

final_score:                       0.50474
genome_localization_score:         0.05462795          (0.4771)
chromosome_localization_score:     0.071284182         (0.42006)
cnv_score:                         0.1816       (0.666666666666667)
translocation_score:               0.156627149620734       (0.57498953605262)
insertion_breakpoint_score:        0          (0)
loh_score:                         0          (0)
tp53_score:                        0          (0)

mutation_density_of_region:        7.813263e-07
mutation_density_of_chromosome:    8.3654416848903e-08
standard_deviations_from_mean_of_chromosome_mutation_density:     0.638884763760033

density_of_copy_number_switches:   2.929974e-07
number_of_aberrant_copy_number_states:    1
aberrant_copy_number_states:
        0:        9

density_of_translocation_breakpoints:   4.88328938372888e-07
number_of_intertranslocational_chromosomes:        2
intertranslocational_chromosomes:
        9:        3
        20:       2

tp53_mutation_present:    0
---
```
