# Supplementary figures and images for: ShatterProof: operational detection and quantification of chromothripsis
Source: BMC Bioinformatics. 2014 Mar 19;15:78. doi: 10.1186/1471-2105-15-78 (PMC3999944; doi:10.1186/1471-2105-15-78)

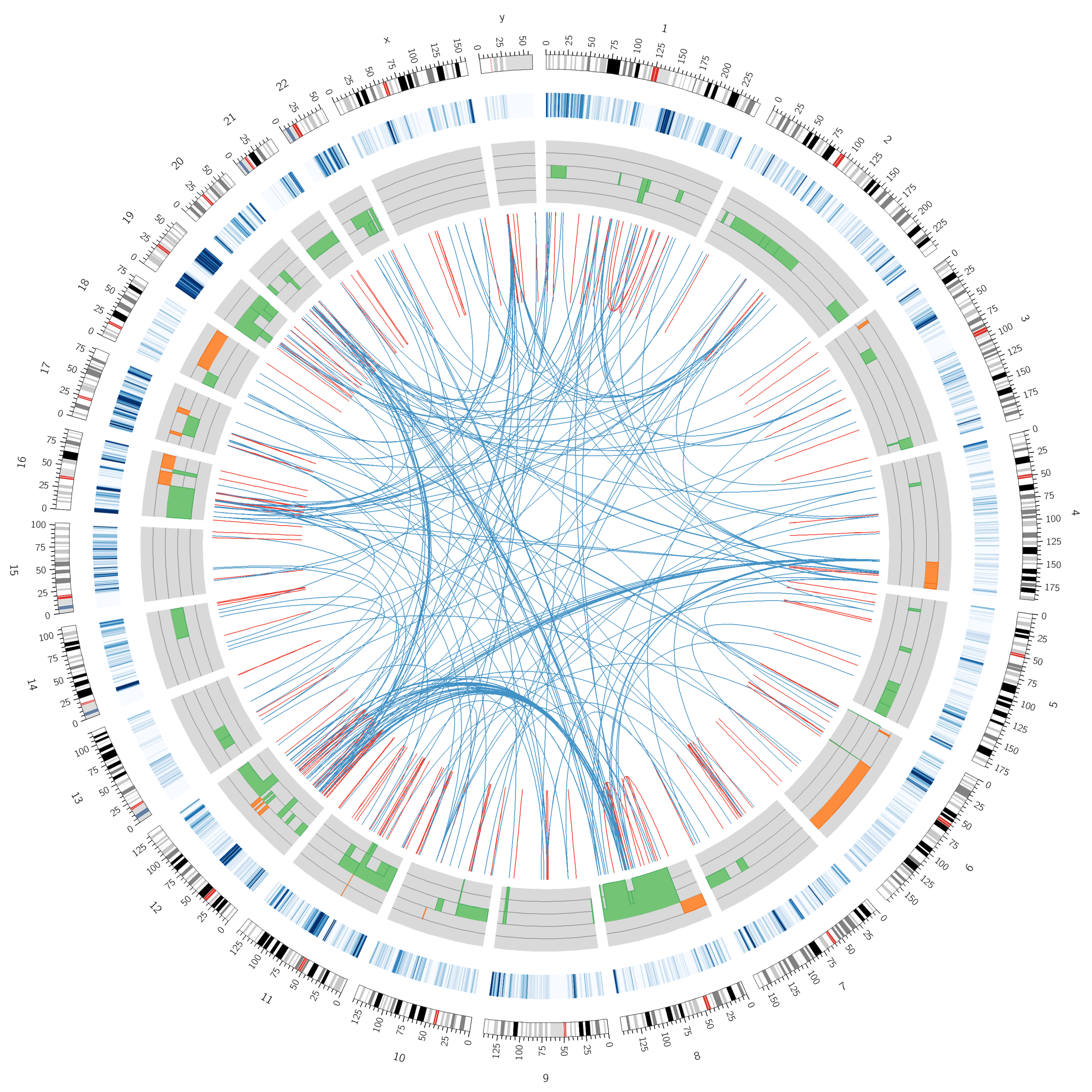

Supplement: Additional file 5 — Supplementary Figure 2 - Clustering of SVs to chromosome 4,8,12, and 20 of prostate adenocarcinoma genome (LTL-1). Circos plot of prostate cancer adenocarcinoma genome (sample LTL-1). From outermost ring going inward each ring indicates: cytogenetic bands, genetic density, histogram of CNV locations, and link diagram of translocation data (interchromosomal in blue, intrachromosomal in red). The plot demonstrates clustering of structural variation to chromosomes 4,8,12, and 20. [file 1471-2105-15-78-S5.pdf]

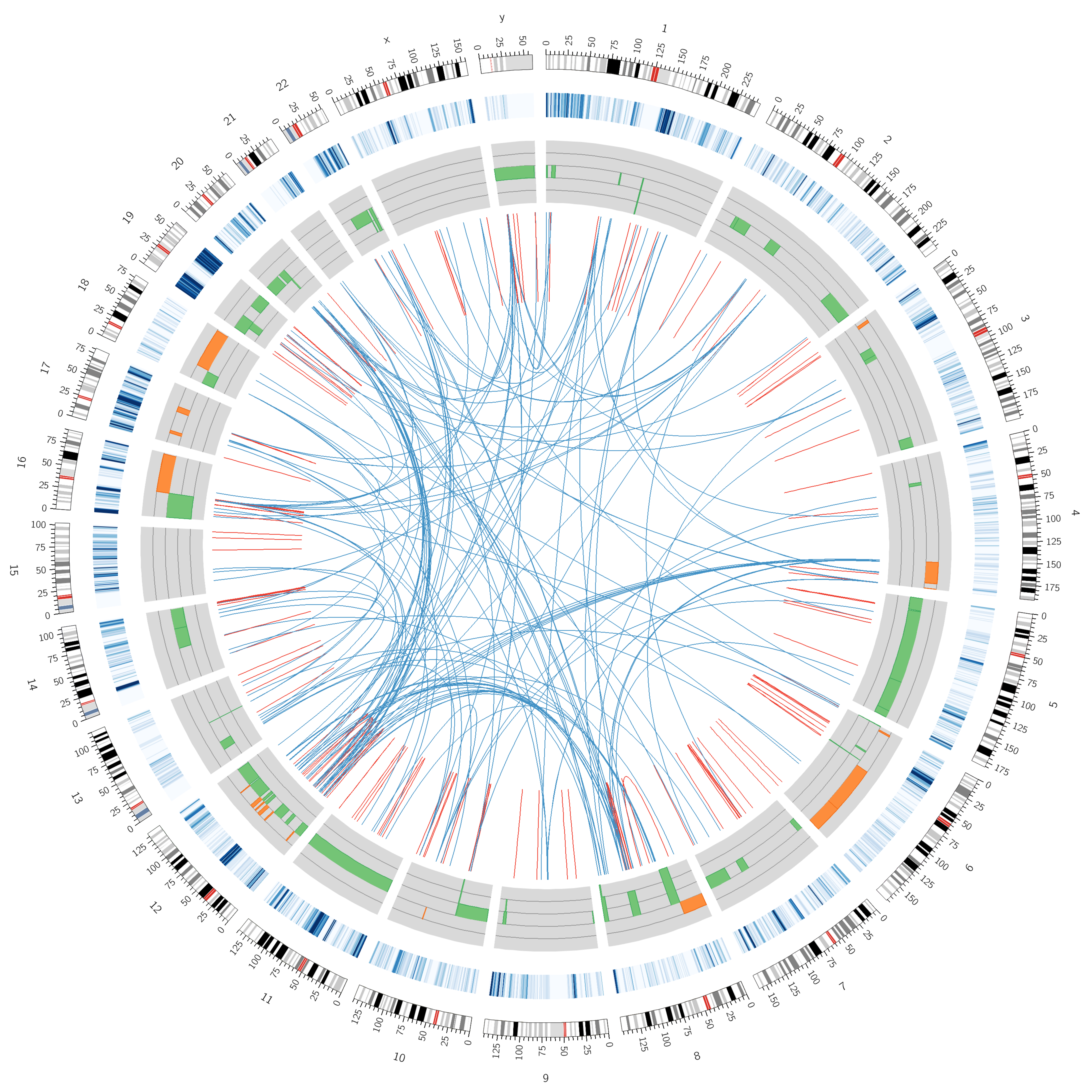

Supplement: Additional file 6 — Supplementary Figure 3 - Clustering of SVs to chromosome 4,8,12, and 20 of prostate adenocarcinoma genome (LTL-2). Circos plot of prostate cancer adenocarcinoma genome (sample LTL-2). From outermost ring going inward each ring indicates: cytogenetic bands, genetic density, histogram of CNV locations, and link diagram of translocation data (interchromosomal in blue, intrachromosomal in red). The plot demonstrates clustering of structural variation to chromosomes 4,8,12, and 20. [file 1471-2105-15-78-S6.pdf]

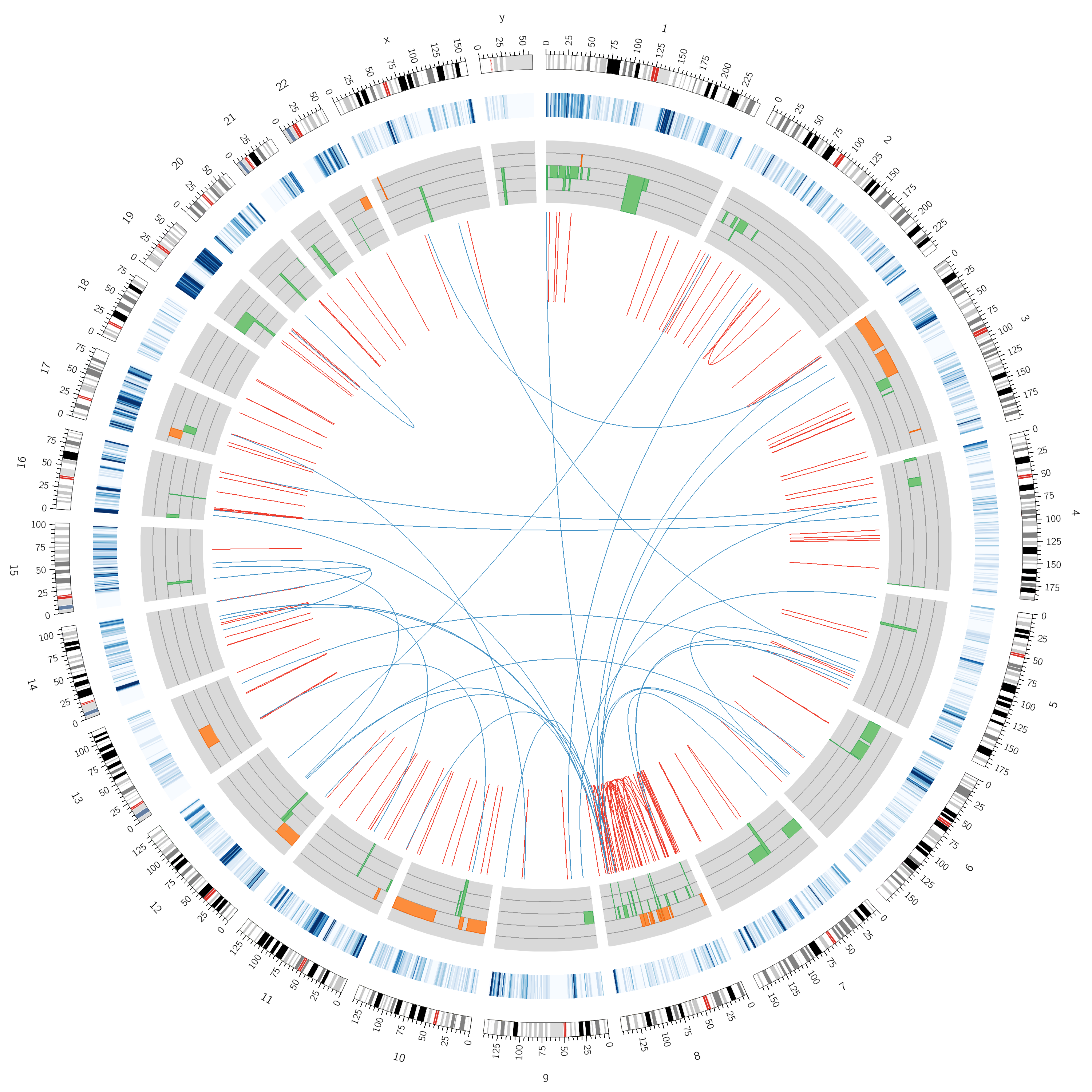

Supplement: Additional file 7 — Supplementary Figure 4 - Clustering of SVs to chromosome 15 of colorectal cancer genome. Circos plot of colorectal cancer genome. From outermost ring going inward each ring indicates: cytogenetic bands, genetic density, histogram of CNV locations, and link diagram of translocation data (interchromosomal in blue, intrachromosomal in red). The plot clearly demonstrates a clustering of structural variation to chromosome 15. [file 1471-2105-15-78-S7.pdf]

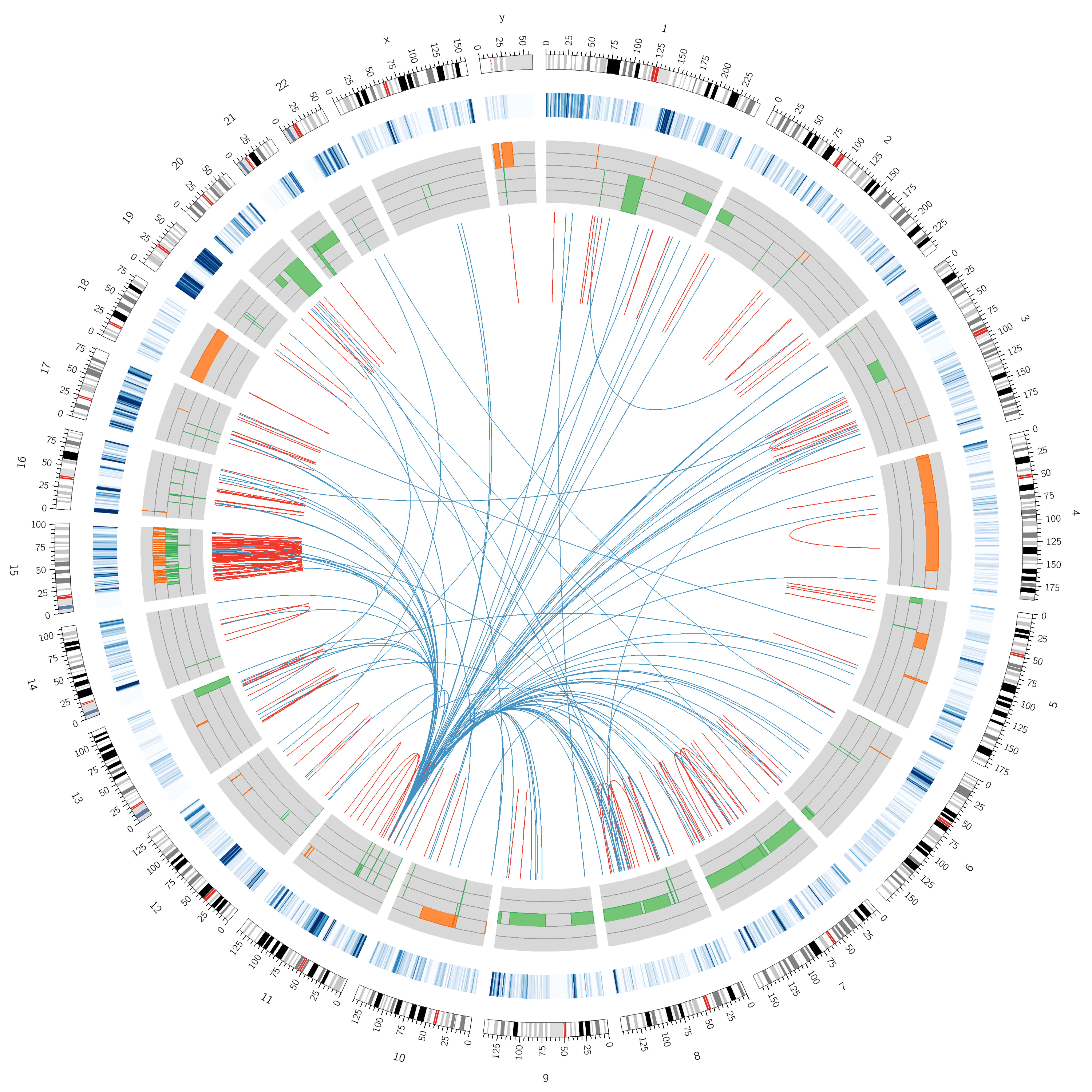

Supplement: Additional file 8 — Supplementary Figure 5 - Clustering of SVs to chromosome 8 of SCLC genome. Circos plot of SCLC genome. From outermost ring going inward each ring indicates: cytogenetic bands, genetic density, histogram of CNV locations, and link diagram of translocation data (interchromosomal in blue, intrachromosomal in red). The plot clearly demonstrates a clustering of structural variation to chromosome 8. [file 1471-2105-15-78-S8.pdf]

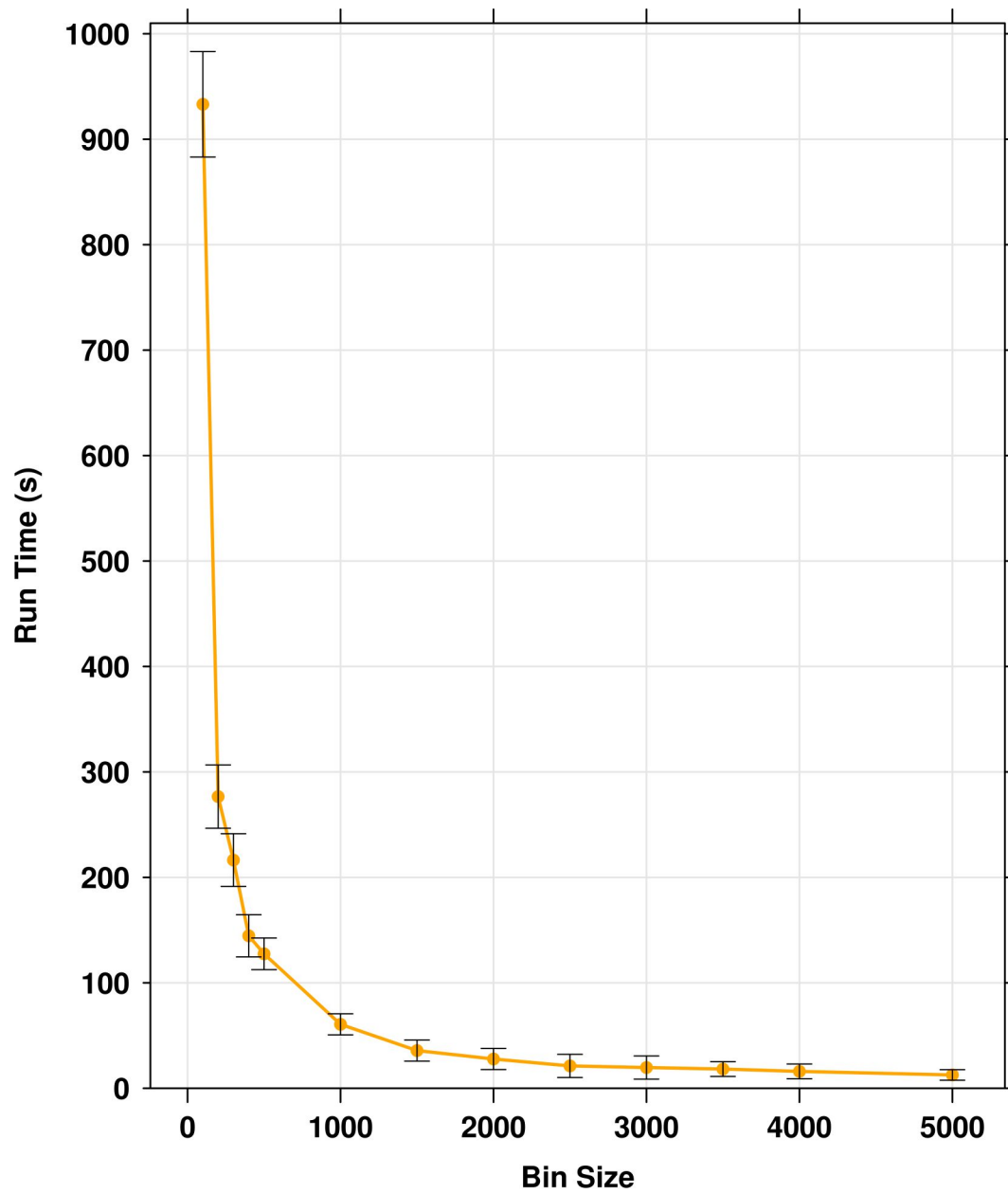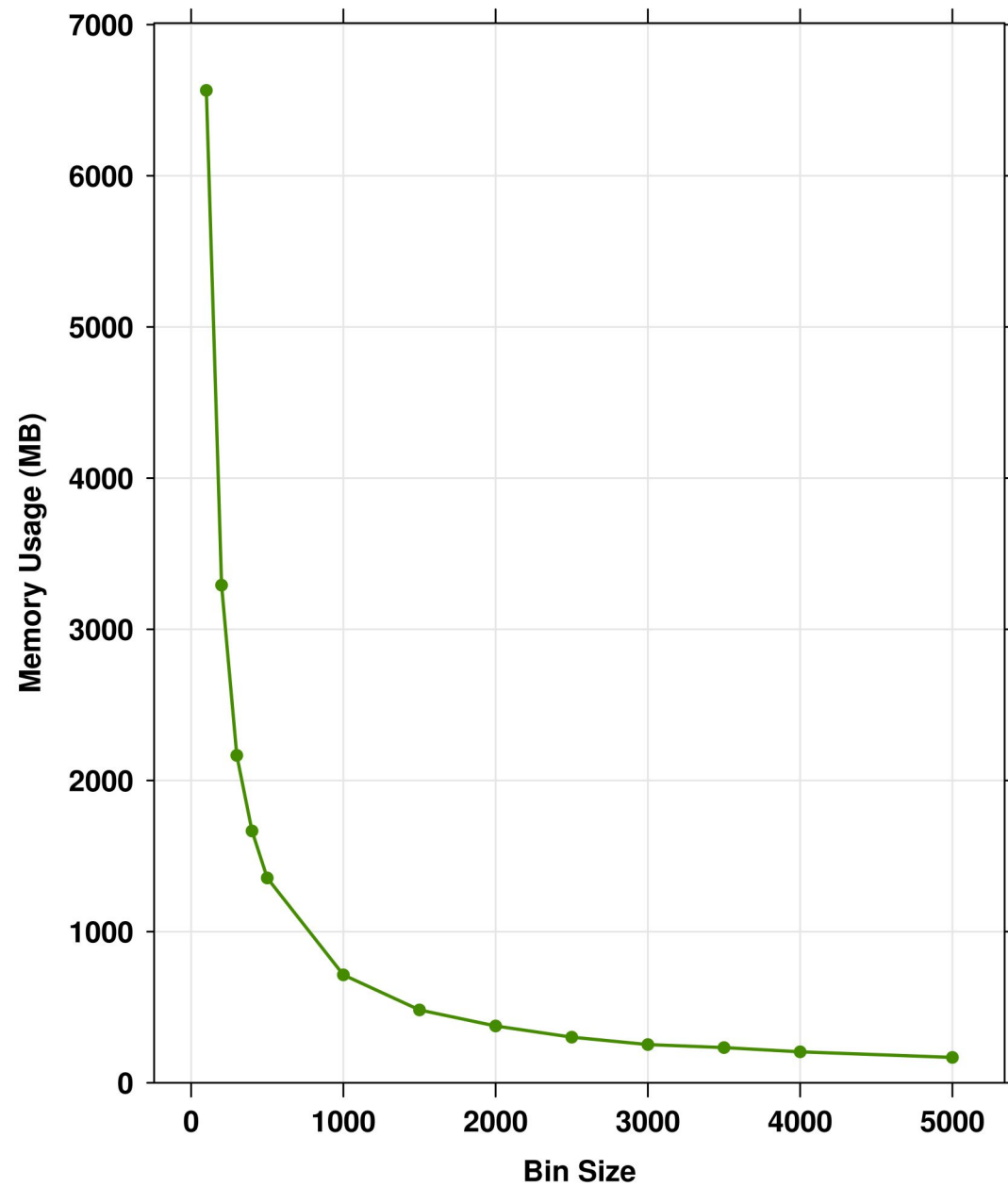

Supplement: Additional file 10 — Supplementary Figure 6 - Run time and memory consumption. These plots illustrate the inverse relationship between run time/memory consumption and bin size. The error bars on the run time vs bin size plot indicate the range of times that were observed across 10 trial runs. No error bars are present on the memory consumption vs bin size plot as we found memory consumption to be consistent between trial runs using the same bin size. [file 1471-2105-15-78-S10.pdf]
