# Supplementary material for: ShatterProof: operational detection and quantification of chromothripsis
Source: BMC Bioinformatics. 2014 Mar 19;15:78. doi: 10.1186/1471-2105-15-78 (PMC3999944; doi:10.1186/1471-2105-15-78)

Primer Design

Primers were designed using the NCBI Primer Blast, (http://www.ncbi.nlm.nih.gov/tools/primer-blast/index.cgi?LINK_LOC=BlastHome) with the parameters set to create a product of 150-500bp within the region surrounding the identified translocation. Primers were not permitted to be within 25 nucleotides of the predicted breakpoint. Primers were synthesized by Integrated DNA Technologies (Coralville, USA) and dissolved in water. Primer working stocks contained both forward and reverse primers at a concentration of 10 µM.

PCR reactions

PCR reactions were carried out in 96 well plates (Life Technologies, Mississauga, Canada) using a C1000 thermocycler (Bio-Rad Laboratories, Mississauga, Canada). Each reaction contained 10 µl of Q5 master mix (New England Biolabs, Whitby, Canada) with 1ng of template DNA, 2 µl of primer working stock mix and water to adjust the final volume to 20 µl. Reactions were heated to 98°C for 2 minutes followed by 35 cycles of 98°C for 30 seconds, 64°C for 20 seconds and 72°C for 30 seconds. Following cycling, the reactions were heated at 72°C for 5 minutes and then held at 12°C until used for electrophoresis.

Electrophoresis

Loading dye (4 µl) was added to the entire reaction mix and the samples were electrophoresed on a 1% agarose gel containing 1:10,000 dilution of SYBR Safe gel stain at 240V in Tris-Acetate EDTA electrophoresis buffer for 40 minutes. Samples were electrophoresed along with 2 µg of 1 kb+ marker (Life Technologies). The gel was photographed with an Alpha Innotech MultiImage III (Fisher Scientific, Ottawa, Canada) gel documentation unit.

Tumor Sample	Expected	Observed	Observed in Reference	Primer sequence F	Primer sequence R
CPCG05_CHR3:151148544 - CHR5:39787751	250	yes	yes	CACCAGCACTACAGGCAAGA	AAATCACCGGTCTTCTGCGT
CPCG05_CHR3:151148544 - CHR5:39787751	368	no	no	TCACTGAGTTAGGTAGGTGCTAGA	AACACTGTCTCGATCATGCTGT
CPCG05_CHR11: 38812669 – CHR8:52730251	427	yes	yes	AAGGAATTCCCTACCAGAACATT	TCAGCAACCTCAGACCAACC
CPCG05_CHR8:52730251 - CHR11:38812669	322	multiple bands	no	TCCATAGGTGTACTGAGATTGCC	ACCCTCATACATATGCAGCCTC
CPCG05_CHR1:33516335-CHR7:24448712	272	yes	no	TACGCATCATGATCTAATGAGCCA	CAGTACCCCGGCTGCTAGTC
CPCG05_CHR7:24448712 - CHR1:33516335	368	yes	no	CCCACAATGACTGCAGATAAAATC	GCTATGTTTCCCAGTACCCCG
CPCG19_CHR13: 74313862 – CHR8: 15289367	227	yes	yes	CCAAGAATGTGCTAAACCCAGA	GCCTGGAATGCAAAGATAGTGC
CPCG19_CHR8: 15289367 – CHR13: 74313862	440	yes	no	GCCTGGAATGCAAAGATAGTGC	GCCTGGAATGCAAAGATAGTGC
CPCG19_CHR8: 30105890 – CHR1: 168420929	209	yes	no	GGGCAAAGGCCGAACTAACT	CCCCTCCCACCCAAAAATGT
CPCG19_CHR1: 168420929 – CHR8: 30105890	364	multiple bands	no	AGTGACCAGGTTTCTAGCCCA	GGAGCAGTTTAGGGAGGGTC
CPCG19_CHR7: 61778466 – CHR3: 89509517	379	yes	no	AGGTTGCCTCCGTTGTGTAA	TACTCCTTTCTATGCTGCAAGT
CPCG19_CHR3: 89509517 – CHR7: 61778466	208	no	no	TACTCCTTTCTATGCTGCAAGT	AAGGGCCATGATCCCTGATG
CPCG20_CHR10: 55983281 – CHR4: 163863475	248	yes	yes	CGTTTTAGCCGGCATGGTC	TAGGTCAGGCATCCACATTTC
CPCG20_CHR4: 163863475 – CHR10:55983281	255	yes	yes	CCGTTTTAGCCGGCATGGTC	GGCATCCACATTTCCTTCAATTTAC
CPCG40_CHR7: 20394028 – CHR15: 26925537	315	yes	no	TCCCAGGAACTGGTAGTCTAGT	AGCCTGCACAGCAGATTTCA
CPCG40_CHR15: 26925537 – CHR7: 20394028	480	no	no	AGGTCCTGAAGAAGCCTGAG	GAAGCCGAGGCTAAACACAA
CPCG40_CHR11: 38812669 – CHR8: 52730250	149	no	no	ATGTTCTAACAGATTAACAAATACA	GCCTGTTAAATCATTGGAAGAC
CPCG40_CHR8: 52730250 – CHR11: 38812669	321	multiple bands	no	TCCATAGGTGTACTGAGATTGCC	ACCCTCATACATATGCAGCCTC
CPCG40_CHR5: 96521626 - CHR3: 143512459	235	yes	no	TGACCCTGTCCTTACAAGTTGC	TGTAACTACAGCCTCTCATTCG
CPCG40_CHR3: 143512459 - CHR5: 96521626	216	no	no	ATAATTTGGCAGCATTGTCCAAGA	ACAAACTGCTTGAAGATGGTGAT
CPCG40_CHR7: 61778466 - CHR3: 89509517	279	no	yes	TGCATTGTATGCTTTTCCATTATT	ACTCCTTTCTATGCTGCAAGTTAAT
CPCG40_CHR3: 89509517 - CHR7: 61778466	266	multiple bands	no	TCACAGAATTTCCACTAGCCAGG	AATTCCTCCCAAGCAGACCC
CPCG40_CHR4: 95518746 - CHR7: 46904665	178	yes	no	GTCGTCAAGGGTGATGGTGAAAC	GGGCAGGTGTGAGTATGTTCTT
CPCG40_CHR7: 46904665 - CHR4: 95518746	193	multiple bands	no	GCATCTGGTGATGGTCTCAGG	CATACCCTGAGCGGTAAGTCA

Blood Sample	Expected	Observed	Observed in Reference	Primer sequence F	Primer sequence R
CPCG05_CHR3:151148544 - CHR5:39787751	250	yes-faint	yes	CACCAGCACTACAGGCAAGA	AAATCACCGGTCTTCTGCGT
CPCG05_CHR3:151148544 - CHR5:39787751	368	no	no	TCACTGAGTTAGGTAGGTGCTAGA	AACACTGTCTCGATCATGCTGT
CPCG05_CHR11: 38812669 – CHR8:52730251	427	yes - faint	yes	AAGGAATTCCCTACCAGAACATT	TCAGCAACCTCAGACCAACC
CPCG05_CHR8:52730251 - CHR11:38812669	322	no	no	TCCATAGGTGTACTGAGATTGCC	ACCCTCATACATATGCAGCCTC
CPCG05_CHR1:33516335-CHR7:24448712	272	yes -faint	no	TACGCATCATGATCTAATGAGCCA	CAGTACCCCGGCTGCTAGTC
CPCG05_CHR7:24448712 - CHR1:33516335	368	no	no	CCCACAATGACTGCAGATAAAATC	GCTATGTTTCCCAGTACCCCG
CPCG19_CHR13: 74313862 – CHR8: 15289367	227	yes	yes	CCAAGAATGTGCTAAACCCAGA	GCCTGGAATGCAAAGATAGTGC
CPCG19_CHR8: 15289367 – CHR13: 74313862	440	no	no	GCCTGGAATGCAAAGATAGTGC	GCCTGGAATGCAAAGATAGTGC
CPCG19_CHR8: 30105890 – CHR1: 168420929	209	yes	no	GGGCAAAGGCCGAACTAACT	CCCCTCCCACCCAAAAATGT
CPCG19_CHR1: 168420929 – CHR8: 30105890	364	no	no	AGTGACCAGGTTTCTAGCCCA	GGAGCAGTTTAGGGAGGGTC
CPCG19_CHR7: 61778466 – CHR3: 89509517	379	yes	no	AGGTTGCCTCCGTTGTGTAA	TACTCCTTTCTATGCTGCAAGT
CPCG19_CHR3: 89509517 – CHR7: 61778466	208	no	no	TACTCCTTTCTATGCTGCAAGT	AAGGGCCATGATCCCTGATG
CPCG20_CHR10: 55983281 – CHR4: 163863475	248	yes	yes	CGTTTTAGCCGGCATGGTC	TAGGTCAGGCATCCACATTTC
CPCG20_CHR4: 163863475 – CHR10:55983281	255	yes	yes	CCGTTTTAGCCGGCATGGTC	GGCATCCACATTTCCTTCAATTTAC
CPCG40_CHR7: 20394028 – CHR15: 26925537	315	no	no	TCCCAGGAACTGGTAGTCTAGT	AGCCTGCACAGCAGATTTCA
CPCG40_CHR15: 26925537 – CHR7: 20394028	480	no	no	AGGTCCTGAAGAAGCCTGAG	GAAGCCGAGGCTAAACACAA
CPCG40_CHR11: 38812669 – CHR8: 52730250	149	no	no	ATGTTCTAACAGATTAACAAATACA	GCCTGTTAAATCATTGGAAGAC
CPCG40_CHR8: 52730250 – CHR11: 38812669	321	no	no	TCCATAGGTGTACTGAGATTGCC	ACCCTCATACATATGCAGCCTC
CPCG40_CHR5: 96521626 - CHR3: 143512459	235	no	no	TGACCCTGTCCTTACAAGTTGC	TGTAACTACAGCCTCTCATTCG
CPCG40_CHR3: 143512459 - CHR5: 96521626	216	no	no	ATAATTTGGCAGCATTGTCCAAGA	ACAAACTGCTTGAAGATGGTGAT
CPCG40_CHR7: 61778466 - CHR3: 89509517	279	yes	yes	TGCATTGTATGCTTTTCCATTATT	ACTCCTTTCTATGCTGCAAGTTAAT
CPCG40_CHR3: 89509517 - CHR7: 61778466	266	no	no	TCACAGAATTTCCACTAGCCAGG	AATTCCTCCCAAGCAGACCC
CPCG40_CHR4: 95518746 - CHR7: 46904665	178	yes	no	GTCGTCAAGGGTGATGGTGAAAC	GGGCAGGTGTGAGTATGTTCTT
CPCG40_CHR7: 46904665 - CHR4: 95518746	193	no	no	GCATCTGGTGATGGTCTCAGG	CATACCCTGAGCGGTAAGTCA

Prostate Tumour and Normal Blood DNA

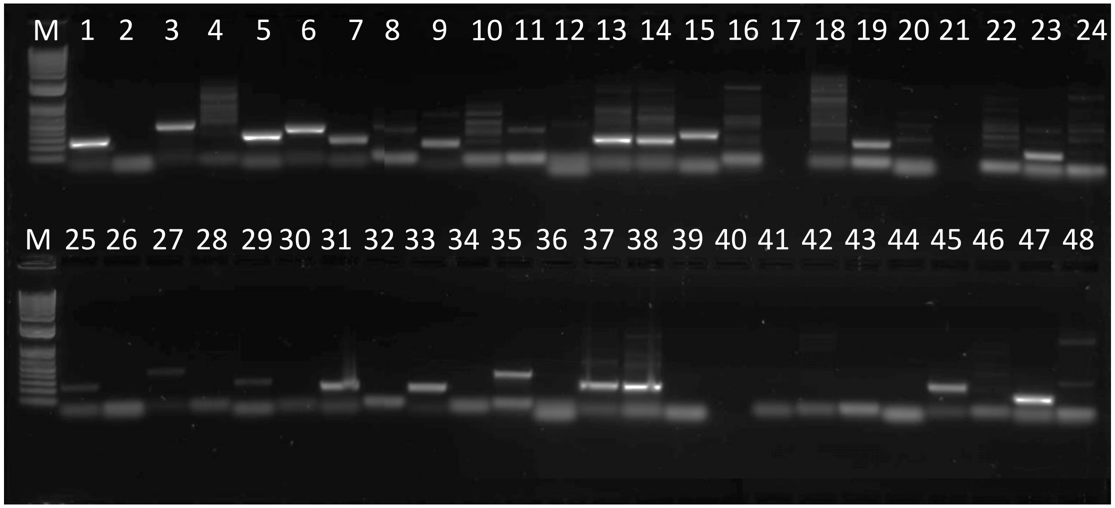

Human Reference DNA

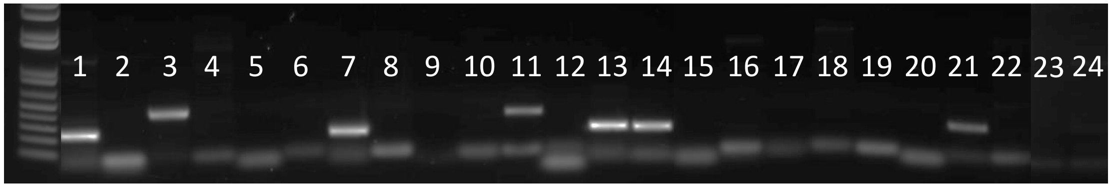

Supplement: Additional file 9 — PCR validation methods and data. This document contains the methods used to perform PCR validation on a subset of the translocation calls presented in the paper as well as the specific primer sequences and the validation results. [file 1471-2105-15-78-S9.pdf]
